# Assessment of Underuse and Overuse of Screening Tests for Co-occurring Conditions Among Children With Obesity

**DOI:** 10.1001/jamanetworkopen.2022.22101

**Published:** 2022-07-14

**Authors:** Mona Sharifi, Alyson B. Goodman, Kao-Ping Chua

**Affiliations:** 1 Department of Pediatrics, Section of General Pediatrics, Yale School of Medicine, New Haven, Connecticut; 2 Department of Biostatistics, Health Informatics, Yale School of Public Health, New Haven, Connecticut; 3 Division of Nutrition, Physical Activity, and Obesity, National Center for Chronic Disease Prevention and Health Promotion, Centers for Disease Control and Prevention, Atlanta, Georgia; 4 Department of Pediatrics, Susan B. Meister Child Health Evaluation and Research Center, University of Michigan Medical School, Ann Arbor; 5 Department of Health Management and Policy, University of Michigan School of Public Health, Ann Arbor

## Abstract

**Importance:**

The American Academy of Pediatrics (AAP) recommends laboratory screening for diabetes, dyslipidemia, and liver disease every 2 years among children aged 10 years and older with obesity. Routine screening for hypothyroidism or hyperinsulinemia is not recommended. National data on rates of adherence to AAP-recommended screening and rates of potentially unnecessary endocrine testing are unavailable.

**Objective:**

To assess rates of AAP-adherent screening and potentially unnecessary endocrine testing among privately and publicly insured children diagnosed with obesity at well-child visits.

**Design, Setting, and Participants:**

This cross-sectional study used data from the 2018-2019 IBM MarketScan Commercial Database (which contains claims from privately insured patients across the US) and the 2018-2019 IBM MarketScan Multi-State Medicaid Database (which contains claims from Medicaid patients in multiple states). A total of 156 773 children aged 10 to 18 years with obesity diagnosis codes at a well-child visit from December 1, 2018, through November 30, 2019, were included. Analysis was performed from May 1 to October 31, 2021.

**Main Outcomes and Measures:**

Primary outcomes were the proportion of children who received AAP-adherent screening during the 729 days before to 30 days after a well-child visit and the proportion of children who received 1 or more potentially unnecessary thyroid or insulin tests during the same period. Proportions of children by payer type were compared using logistic regression models adjusted for age and sex. To assess screening that was potentially ordered by primary care clinicians, the proportion of children without AAP-adherent screening before visits who received this screening within 30 days was calculated.

**Results:**

Of 156 773 children, the mean (SD) age was 13.5 years (2.5) years; 83 305 (53.1%) were male, and 97 595 (62.3%) were publicly insured. Among publicly insured children, 13 167 (13.5%) were Hispanic or Latino, 34 792 (35.6%) were non-Hispanic Black, 42 029 (43.1%) were non-Hispanic White, 3850 (3.9%) were of other race and/or ethnicity (specific races and ethnicities were not available in the database), and 3757 (3.8%) were of unknown race and ethnicity. Race and ethnicity were not available for privately insured children because these data are not reported in the commercial claims database. During the 729 days before to 30 days after well-child visits, 42 849 patients (27.3%) received AAP-adherent screening, including 13 939 (23.6%) of 59 178 privately insured children and 28 910 (29.6%) of 97 595 publicly insured children (average marginal effect for public insurance: 6.8 [95% CI, 6.3-7.2] percentage points). In addition, 46 592 children (29.7%) received potentially unnecessary thyroid or insulin tests, including 12 834 (21.7%) of 59 178 privately insured children and 23 198 (23.8%) of 97 595 publicly insured children (average marginal effect for public insurance: 2.4 [95% CI, 2.0-2.8] percentage points). Among 129 104 children who did not receive AAP-adherent screening before visits, 15 090 (11.7%) received this screening within 30 days.

**Conclusions and Relevance:**

In this cross-sectional study, similar proportions of children diagnosed with obesity at well-child visits received recommended and nonrecommended screening tests for obesity-related co-occurring conditions, suggesting that specific actionable guidelines and interventions are needed to prevent overuse and underuse of screening tests among children with obesity.

## Key Points

**Question:**

Among children aged 10 to 18 years diagnosed with obesity at well-child visits, what proportion received laboratory screening tests recommended by the American Academy of Pediatrics for obesity-related conditions, and what proportion received potentially unnecessary screening tests for hypothyroidism or hyperinsulinemia?

**Findings:**

In this national cross-sectional analysis of 156 773 children in 2018-2019 commercial and Medicaid claims databases, 27.3% of children diagnosed with obesity at well-child visits received laboratory screening that was adherent to American Academy of Pediatrics guidelines, and 23.0% received potentially unnecessary thyroid or insulin testing.

**Meaning:**

This study’s findings suggest that specific actionable guidelines and interventions are needed to prevent overuse and underuse of screening tests for children with obesity.

## Teaser

This cross-sectional study uses data from commercial and Medicaid claims databases to assess rates of American Academy of Pediatrics–adherent screening and potentially unnecessary endocrine testing among privately and publicly insured children aged 10 to 18 years who received a diagnosis of obesity at well-child visits.

## Introduction

Preventing pediatric obesity-associated complications is a public health priority.^1^ Among children and adolescents with overweight and obesity, diabetes occurs in approximately 21%, dyslipidemia in approximately 39% to 49%, and nonalcoholic fatty liver disease in approximately 26%.^2,3,4,5^ In 2007, the American Academy of Pediatrics (AAP) released expert committee recommendations for the assessment of child and adolescent overweight and obesity, revising guidelines originally issued in 1998.^6,7^ For children aged 10 years and older with obesity, the recommendations include laboratory screening every 2 years for diabetes, dyslipidemia, and nonalcoholic fatty liver disease.^6^ The recommendations discourage routine screening for thyroid dysfunction in the absence of abnormal linear growth and do not include insulin testing, which is not recommended for diagnosis of diabetes.^6,8,9^

Although evidence from single institutions and clinician self-reporting suggests that rates of screening for co-occurring conditions among children with obesity are low,^10,11,12,13,14,15,16,17,18,19,20^ no study, to our knowledge, has used national data to assess adherence to AAP recommendations and the use of potentially unnecessary screening for hypothyroidism or hyperinsulinemia. Furthermore, it is unknown whether the use of screening tests for co-occurring conditions among children with obesity differs among children with public vs private insurance. Publicly insured children might be less likely to receive appropriate screening and/or more likely to receive unnecessary screening than those with private insurance because of systematic disparities in access to high-quality preventive services. Children with private insurance might experience financial barriers to screening owing to out-of-pocket costs and copayments associated with laboratory testing. Identifying differences in the use of screening tests among children with private vs public insurance children could aid in determining whether quality improvement efforts should be broadly targeted or tailored to settings and clinicians with specific payer mixes.

Well-child visits for children with obesity represent important opportunities for primary care clinicians to ensure AAP-adherent screening is provided, but these visits also represent opportunities to order unnecessary testing. In this cross-sectional study, we used national claims data to identify privately and publicly insured children aged 10 to 18 years who were diagnosed with obesity at well-child visits from December 1, 2018, to November 30, 2019. Among these children, we calculated the proportion with AAP-adherent screening and potentially unnecessary insulin or thyroid testing during the 2 years before to 30 days after the visit. Among children due to receive AAP-recommended screening at visits, we assessed the proportion who received AAP-adherent screening within 30 days and the proportion who received potentially unnecessary insulin or thyroid testing within 30 days, both of which were potential signs of screening that may have been ordered by primary care clinicians. In addition, we compared rates of screening by payer type and assessed whether the tests used to achieve AAP-adherent screening were parsimonious (ie, the minimum number of tests were ordered to achieve AAP-adherent screening) or overly broad (ie, additional potentially unnecessary tests were ordered in conjunction with recommended tests). Findings from this study could inform the content of future guidelines and interventions to improve screening for co-occurring conditions among children with obesity.

## Methods

### Data Source

From May 1 to October 31, 2021, we conducted a cross-sectional analysis of data from the 2018-2019 IBM MarketScan Commercial Database (commercial database) and the 2018-2019 IBM MarketScan Multi-State Medicaid Database (Medicaid database). The commercial database includes claims from enrollees younger than 65 years with private insurance from medium to large employers across the US; annual sample sizes range from 27 to 29 million enrollees.^21^ The Medicaid database includes claims from enrollees from multiple unidentified states with insurance coverage from Medicaid or the Children’s Health Insurance Program; annual sample sizes range from 10 to 12 million enrollees. Data elements included diagnosis codes from the *International Statistical Classification of Diseases, Tenth Revision, Clinical Modification *(*ICD-10-CM*) and procedure codes from the *Current Procedural Terminology* system. Data from 2017 were used as a lookback period to assess testing and diagnosis codes before well-child visits. The study was approved by the institutional review board of the University of Michigan Medical School, which determined the study was exempt from human participant review, with a waiver of informed consent granted because of the use of deidentified data. This study followed the Strengthening the Reporting of Observational Studies in Epidemiology (STROBE) reporting guideline for cross-sectional studies.

### Sample

Children were included if they were aged 10 to 18 years and had a well-child visit claim between December 1, 2018, and November 30, 2019, with an *ICD-10-CM* diagnosis code for obesity on the claim (codes are provided in eTable 1 and eTable 2 in the Supplement). The index date was the date of the earliest well-child visit claim during the study period. To ensure the ability to observe both previous and immediate testing after the well-child visit, we excluded children without continuous enrollment during a measurement period from 729 days before the index date (2 years minus 1 day) to 30 days after the index date. We chose to examine testing within the previous 729 days because a patient who received obesity screening tests 730 days or more before the index date should have received testing to be adherent to AAP recommendations. We examined testing within 30 days after the index date to account for tests that were ordered but delayed in completion. Because 1 analysis assessed screening that was potentially ordered by primary care clinicians, we purposefully limited the sample to children who had well-child visits during which obesity was diagnosed. These children represented a subset of all children with obesity because many children do not receive well-child visits, and obesity is frequently undercoded.^12,14,19^

We categorized patient age ranges as 10 to 12 years, 13 to 15 years, and 16 to 18 years and biological sex as male and female. Race and ethnicity data were only available in the Medicaid database and reflected self-report or proxy report in response to an optional question at Medicaid enrollment. Race and ethnicity are categorized in the database as Hispanic or Latino, non-Hispanic Black, non-Hispanic White, other (specific races and ethnicities included in this category were not available in the database), and unknown. Payer type was defined as public if data were derived from the Medicaid database and private if data were derived from the commercial database.

### Obesity Screening Tests

We searched outpatient files for claims with *Current Procedural Terminology *codes corresponding to 6 groups of blood tests (eTable 3 in the Supplement): (1) tests that evaluated the level of glucose but not hepatic transaminases, including a test of glucose alone, a basic metabolic panel, and a renal function panel; (2) tests that evaluated the level of glucose and both hepatic transaminases, including a comprehensive metabolic panel and a general health panel, the latter of which comprised a comprehensive metabolic panel, complete blood count, and thyroid-stimulating hormone test; (3) tests that evaluated 1 or more hepatic transaminase levels but not glucose, including a hepatic function panel, a test of alanine aminotransferase (ALT) alone, or a test of aspartate aminotransferase (AST) alone; (4) a lipid panel or individual components of a lipid panel, including tests of total cholesterol, high-density lipoprotein cholesterol, low-density lipoprotein cholesterol, and very low-density lipoprotein cholesterol; (5) tests that evaluated thyroid function or insulin level, including thyroid-stimulating hormone, free or total thyroxine, and free or total insulin; and (6) other diabetes screening tests, including glucose tolerance and hemoglobin A_1c_ tests.

### Measures Assessing Underuse and Overuse of Screening Tests

We created 4 measures to assess AAP-adherent screening and potentially unnecessary endocrine testing, both overall (regardless of which clinician ordered the tests) and specifically for testing that was likely ordered by primary care clinicians at the well-child visit. These measures comprised (1) the overall proportion of children who received AAP-adherent screening, (2) the overall proportion of children who received potentially unnecessary thyroid or insulin testing, (3) the 30-day rate of AAP-adherent screening among children who did not receive this screening before the well-child visit, and (4) the 30-day rate of potentially unnecessary thyroid or insulin testing among children who did not receive AAP-adherent screening before the well-child visit.

#### Overall Proportion With AAP-Adherent Screening

This measure evaluated the proportion of children in the total sample who received AAP-adherent screening during the 729 days before to 30 days after the index visit. The AAP recommendations advise laboratory testing to evaluate children aged 10 years and older with obesity for co-occurring conditions every 2 years using a test of fasting glucose, a test of hepatic transaminases, and a lipid panel. We operationalized these recommendations as the occurrence of 1 or more tests evaluating glucose, 1 or more tests evaluating AST, 1 or more tests evaluating ALT, and 1 or more lipid panels. A single test (eg, a comprehensive metabolic panel) could satisfy several of these requirements. We allowed any instance of testing to count toward AAP-adherent screening; moreover, for children aged 10 or 11 years, we included testing that occurred at age 8 or 9 years, respectively. For additional context, we identified which combinations of tests were used to achieve AAP-adherent screening.

#### Overall Proportion With Potentially Unnecessary Thyroid or Insulin Testing

 This measure evaluated the proportion of children in the total sample who received 1 or more thyroid function or insulin tests during the 729 days before to 30 days after the index date. Children with diagnosis codes during this period for conditions that might warrant this testing were excluded; these conditions included endocrine disorders (such as diabetes), chromosomal disorders, eating disorders, malnutrition, amenorrhea, irregular menstruation, and autoimmune diseases (eTable 4 in the Supplement).

#### 30-Day Rate of AAP-Adherent Screening

This measure evaluated the rate of AAP-adherent screening on or within 30 days after the index date among children who did not receive AAP-adherent screening during the 1 to 729 days before the index date. Not all tests had to be ordered on or after the index date. For example, if a patient received a lipid panel screening immediately before the visit and a comprehensive metabolic panel on the day of the visit, the patient was included in the group who received AAP-adherent screening on or within 30 days of the index date.

#### 30-Day Rate of Potentially Unnecessary Thyroid or Insulin Testing

This measure evaluated the rate of screening with 1 or more thyroid function or insulins tests within 30 days after the index date among children who did not receive AAP-adherent screening during the 1 to 729 days before the index date. Children with diagnosis codes for conditions that might warrant this testing (eTable 4 in the Supplement) during the 1 to 729 days before the index date were excluded.

### Statistical Analysis

We calculated unadjusted performance on each measure overall and by payer type. To assess adjusted differences by payer type (public vs private), we used logistic regression models controlling for single year of age and sex. To improve interpretability, we calculated the average marginal effect (AME) to quantity the absolute percentage point change in performance assuming all children were publicly vs privately insured and holding age and sex at their observed values.^22^

For the sensitivity analysis, we recalculated the first and third measures, allowing hemoglobin A_1c_ or glucose tolerance tests to satisfy the definition of AAP-adherent screening (in addition to glucose level testing already included in the primary analysis). Hypothesis tests were 2-sided with a significance threshold of α = .05. All analyses were conducted using SAS software, version 9.4 (SAS Institute Inc), and Stata software, version 15.1 MP (StataCorp Ltd).

## Results

### Sample

During the study period, 1 622 605 privately insured patients aged 10 to 18 years had 1 or more well-child visit claims; of those, 96 191 patients (5.9%) had 1 or more well-child visit claims containing a diagnosis code for obesity. We excluded 37 103 children with enrollment lapses, leaving 59 178 privately insured children. Among 1 216 142 publicly insured patients aged 10 to 18 years enrolled at any point during the study period, 139 128 (11.4%) had 1 or more well-child visit claims containing a diagnosis code for obesity. We excluded 41 533 children with enrollment lapses, leaving 97 595 publicly insured children in the sample.

Characteristics of the combined sample of 156 773 children are shown in Table 1. The mean (SD) age was 13.5 (2.5) years; 73 738 children (47.0%) were female, 83 305 (53.1%) were male, and 97 595 (62.3%) were publicly insured. Among publicly insured children, 13 167 (13.5%) were Hispanic or Latino, 34 792 (35.6%) were non-Hispanic Black, 42 029 (43.1%) were non-Hispanic White, 3850 (3.9%) were of other race and/or ethnicity (specific races and ethnicities were not available in the database), and 3757 (3.8%) were of unknown race and ethnicity. Race and ethnicity were not available for privately insured children because these data are not reported in the commercial database.

**Table 1.** Characteristics of Children Aged 10 to 18 Years Who Had a Well-Child Visit With a Diagnosis Code for Obesity in the 2018-2019 IBM MarketScan Commercial and Multi-State Medicaid Databases

Characteristic	Children, No. (%)^a^		
	Overall	Privately insured	Publicly insured
Total children, No.	156 773	59 178	97 595
Age range, y			
10-12	61 697 (39.4)	21 714 (36.7)	40 253 (41.2)
13-15	54 865 (35.0)	20 422 (34.5)	34 443 (35.3)
16-18	39 941 (25.5)	17 042 (28.8)	22 899 (23.5)
Sex			
Male	83 305 (53.1)	32 914 (55.6)	50 121 (51.4)
Female	73 738 (47.0)	26 264 (44.4)	47 474 (48.6)
Race and ethnicity^b^			
Hispanic or Latino	NA	NA	13 167 (13.5)
Non-Hispanic			
Black	NA	NA	34 792 (35.6)
White	NA	NA	42 029 (43.1)
Other^c^	NA	NA	3850 (3.9)
Unknown	NA	NA	3757 (3.8)

Abbreviation: NA, not available.

^a^ Values may not total 100% due to rounding.

^b^ Race and ethnicity data were only available in the MarketScan Multi-State Medicaid Database and reflect patients’ race and ethnicity as recorded by the state.

^c^ Specific races and ethnicities included in this category were not available in the database.

### Overall Screening

Among all 156 773 children, 42 849 (27.3%) received AAP-adherent screening, and 46 592 (29.7%) received potentially unnecessary insulin or thyroid testing during the 729 days before to 30 days after the index date (Table 2). Overall, 13 939 (23.6%) of the 59 178 privately insured children and 28 910 (29.6%) of the 97 595 publicly insured children received AAP-adherent screening (AME for public insurance: 6.8 [95% CI, 6.3-7.2] percentage points); 12 834 of the 59 178 privately insured children (21.7%) and 23 198 of the 97 595 publicly insured children (23.8%) received potentially unnecessary thyroid or insulin testing (AME for public insurance: 2.4 [95% CI, 2.0-2.8] percentage points). Among the 42 849 privately and publicly insured children with AAP-adherent screening, the 2 most common combinations of tests meeting our definition of adherence were a comprehensive metabolic panel plus lipid panel (27 878 children [65.1%]) and a general health panel (complete blood count, comprehensive metabolic panel, and thyroid function test) plus lipid panel (4962 children [11.6%]) (Table 3).

**Table 2.** Screening Tests for Co-occurring Conditions Among Children Aged 10 to 18 Years During the 729 Days Before to 30 Days After a Well-Child Visit With a Diagnosis Code for Obesity

Screening test	Children, No. (%)		
	Overall (n = 156 773)	Privately insured (n = 59 178)	Publicly insured (n = 97 595)
Tests that include glucose but not hepatic transaminases			
Glucose alone	16 292 (10.4)	4574 (7.7)	11 718 (12.0)
Basic metabolic panel	9130 (5.8)	2404 (4.1)	6726 (6.9)
Renal function panel	895 (0.6)	179 (0.3)	716 (0.7)
Tests that include glucose and both hepatic transaminases			
General health panel^a^	9024 (5.8)	6815 (11.5)	2209 (2.3)
Comprehensive metabolic panel	41 962 (26.8)	12 076 (20.4)	29 886 (30.6)
Tests that include ≥1 hepatic transaminase but not glucose			
Hepatic function panel	6504 (4.1)	1866 (3.2)	4638 (4.8)
ALT alone	9864 (6.3)	2305 (3.9)	7559 (7.7)
AST alone	8157 (5.2)	1610 (2.7)	6547 (6.7)
Lipid panel or individual components of lipid panel			
Lipid panel	66 227 (42.2)	21 957 (37.1)	44 270 (45.4)
Total cholesterol alone	10 000 (6.4)	3805 (6.4)	6195 (6.3)
HDL cholesterol alone	4974 (3.2)	1533 (2.6)	3441 (3.5)
LDL cholesterol alone	675 (0.4)	359 (0.6)	316 (0.3)
Triglycerides alone	588 (0.4)	256 (0.4)	332 (0.3)
Tests for hypothyroidism or hyperinsulinemia			
TSH alone	37 043 (23.6)	10 341 (17.5)	26 702 (27.4)
Thyroxine alone (free or total)	29 977 (19.1)	11 060 (18.7)	18 917 (19.4)
Insulin alone (free or total)	13 419 (8.6)	4629 (7.8)	8790 (9.0)
Other diabetes screening tests			
Glucose tolerance	799 (0.5)	183 (0.3)	616 (0.6)
Hemoglobin A_1c_	50 309 (32.1)	15 475 (26.1)	34 834 (35.7)
None of the tests above	67 055 (42.8)	28 374 (47.9)	38 681 (39.6)
Outcomes			
AAP-adherent screening	42 849 (27.3)	13 939 (23.6)	28 910 (29.6)
≥1 Potentially unnecessary thyroid or insulin test	46 592 (29.7)	12 834 (21.7)	23 198 (23.8)

Abbreviations: AAP, American Academy of Pediatrics; ALT, alanine aminotransferase; AST, aspartate aminotransferase; HDL, high-density lipoprotein; LDL, low-density lipoprotein; TSH, thyroid-stimulating hormone.

^a^ Comprehensive metabolic panel, complete blood count, and TSH test.

**Table 3.** Laboratory Testing Patterns Defined as Adherent to AAP Screening Recommendations Among Children Aged 10 to 18 Years With AAP-Adherent Screening During the 729 Days Before to 30 Days After a Well-Child Visit With a Diagnosis Code for Obesity

Laboratory testing pattern^a^	Children, No. (%)		
	Overall (n = 42 849)	Privately insured (n = 13 939)	Publicly insured (n = 28 910)
Comprehensive metabolic panel and lipid panel	27 878 (65.1)	6849 (49.1)	21 029 (72.7)
General health panel and lipid panel	4962 (11.6)	4000 (28.7)	962 (3.3)
AST alone, ALT alone, glucose alone, and lipid panel	2988 (7.0)	539 (3.9)	2449 (8.5)
Hepatic function panel, glucose, and lipid panel	912 (2.1)	248 (1.8)	664 (2.3)
Hepatic function panel, basic metabolic panel, and lipid panel	869 (2.0)	111 (0.8)	758 (2.6)
AST alone, ALT alone, basic metabolic panel, and lipid panel	223 (0.5)	30 (0.2)	193 (0.7)
AST alone, ALT alone, renal function panel, and lipid panel	33 (0.10)	6 (0.04)	27 (0.10)
Hepatic function panel, renal function panel, and lipid panel	5 (0.01)	1 (0.01)	4 (0.01)
≥2 Of the above patterns	4979 (11.6)	2155 (15.5)	2824 (9.8)

Abbreviations: AAP, American Academy of Pediatrics; ALT, alanine aminotransferase; AST, aspartate aminotransferase.

^a^ The first 8 categories are mutually exclusive. A patient who met criteria for more than 1 category (eg, comprehensive metabolic panel, lipid panel, and general health panel) was assigned to the ≥2 of the above patterns category.

### Screening Potentially Ordered by Primary Care Clinicians

Among 129 104 children who did not receive AAP-adherent screening before well-child visits, 15 090 (11.7%) received AAP-adherent screening within 30 days, and 12 704 (9.9%) received 1 or more potentially unnecessary thyroid or insulin tests within 30 days. Among 49 981 privately insured and 79 033 publicly insured children, 4748 (9.5%) and 10 353 (13.1%), respectively, received AAP-adherent screening within 30 days (AME for public insurance: 4.0 [95% CI, 3.6-4.3] percentage points), and 4498 (9.0%) and 8219 (10.4%) received 1 or more potentially unnecessary thyroid or insulin tests within 30 days (AME for public insurance: 1.5 [95% CI, 1.1-1.8] percentage points).

### Sensitivity Analysis

Expanding our definition to include hemoglobin A_1c_ or glucose tolerance tests increased the overall proportion of children who received AAP-adherent screening from 27.3% to 28.9% (measure 1). Among children who did not receive AAP-adherent screening during the 1 to 729 days before the index date, the proportion who received AAP-adherent screening within 30 days after the index date increased from 11.7% to 13.6% (measure 3).

## Discussion

In this national cross-sectional study of privately and publicly insured children diagnosed with obesity at well-child visits, only 27.3% of children received AAP-adherent screening for diabetes, nonalcoholic fatty liver disease, and dyslipidemia during the 2 years before to 30 days after visits, whereas 29.7% received potentially unnecessary screening for thyroid dysfunction or hyperinsulinemia during this period. Among children due for AAP-adherent screening at well-child visits, rates of this screening and potentially unnecessary insulin or thyroid testing within 30 days were similar, at 11.7% and 9.9%, respectively. Although screening rates varied by payer type, deficits in appropriate and inappropriate screening were large among both privately and publicly insured children. These results collectively suggest underuse and overuse of screening tests for co-occurring conditions may be common among all children diagnosed with obesity at well-child visits.

To our knowledge, this is the first national study to assess the use of AAP-adherent screening among children with obesity. The low rate of screening could be explained by lack of knowledge about evidence-based recommendations among clinicians, lack of belief that laboratory testing is needed or will have consequences for patient outcomes, lack of time or resources to prioritize integrating these practices into routine workflows, and/or reluctance to subject children to the pain associated with venipuncture.^23^ Addressing potential barriers to screening is important because early detection of diabetes, dyslipidemia, and nonalcoholic fatty liver disease as part of the comprehensive evaluation of obesity could potentially prompt initiation of and engagement in treatment, reducing risk of long-term complications.

To our knowledge, this is also the first national study to document potential overuse of screening tests for thyroid dysfunction and hyperinsulinemia among children with obesity. Unnecessary laboratory tests contribute to wasteful health care spending.^24^ For example, the estimated mean price of a thyroid-stimulating hormone test is $75.^25^ In addition, potential false-positive results could yield downstream unnecessary spending and distress from follow-up testing and visits.

Among children who received AAP-adherent screening, laboratory tests were often overly broad. The most common combination of laboratory orders to achieve adherence was a lipid panel with either a comprehensive metabolic panel or a general health panel. The use of these panels, rather than tests evaluating only transaminases and glucose, increases the potential for detecting anomalous yet incidental findings (eg, high alkaline phosphatase level), potentially increasing spending on unnecessary follow-up care. Overly broad testing may occur because of the convenience of ordering a single test that evaluates both transaminases and glucose compared with ordering separate tests evaluating transaminases and glucose. Notably, more recent guidelines suggest using an ALT test alone to screen for nonalcoholic fatty liver disease,^26^ suggesting clinicians who ordered separate AST and ALT tests could have been even more parsimonious.

Several interventions could be considered to address underuse and overuse of screening tests for co-occurring conditions among children with obesity. Clinical decision support tools can improve the diagnosis of obesity and the recommended screening for co-occurring conditions, potentially by reinforcing to clinicians the importance of this screening.^27,28,29,30,31,32^ An order set in the electronic health record that automatically defaults to the most parsimonious set of screening tests might decrease the use of overly broad tests, potentially reducing health care spending. Alerts, either via interruptive popups or in-line nudges, within the electronic health record can prompt clinicians to order recommended testing for children with obesity. Beyond these clinical interventions, guidelines or other recommendations from professional societies could more emphatically discourage routine thyroid testing, explicitly discourage routine insulin testing, and specify the most parsimonious set of tests to order.

Our finding that publicly insured children were more likely to receive potentially unnecessary endocrine testing is consistent with a study^33^ finding that publicly vs privately insured children were slightly more likely to receive unnecessary care. In contrast, our finding that publicly insured children were more likely to receive AAP-adherent screening contrasts with results of a previous study reporting that publicly and privately insured children had similar rates of recommended lipid screening.^34^ Although these payer-based differences were notable, the extent of these differences was small compared with the high absolute frequency of overuse and underuse of screening tests. Therefore, efforts to increase the use of appropriate screening and decrease the use of inappropriate screening may need to include all children while also ensuring equitable care delivery.

Though not the focus of this study, we found that few children aged 10 to 18 years in the commercial and Medicaid claims databases had well-child visits containing an obesity diagnosis code, even though 19.3% of US children in this age group have obesity.^35^ This finding is consistent with those of previous studies reporting that most children with overweight or obesity are undiagnosed.^11,12,14,18,19,20,36,37,38^ Previous studies have found higher rates of diet and lifestyle education, counseling, screening, and referral among children with documented obesity, suggesting the importance of efforts to improve documentation.^11,13,17,18^

### Limitations

This study has limitations. First, the claims databases lacked information on height or weight, so we relied on obesity diagnosis codes to define the study sample. Results may not generalize to children with obesity who do not have a well-child visit or lack a recorded diagnosis of obesity on the visit claim. If the lack of this diagnosis represents either a lack of attention to obesity by the clinician (perhaps as a result of less severe obesity) or serves as a signal of the quality of care, we would expect rates of AAP-adherent screening to be even lower among these children. Second, because the database lacked clinical information and results of laboratory testing, we could not definitely determine whether thyroid or insulin testing was in fact unnecessary. Our estimates likely represent an upper bound of the true prevalence of unnecessary endocrine testing. Third, results might overestimate adherence to AAP-recommended screening because fasting status was unknown, although even a random nonfasting glucose test can screen for diabetes.^8^ Fourth, we could not determine whether screening was performed specifically to assess co-occurring conditions. Fifth, we could only adjust for age and sex when assessing differences by payer type owing to data limitations. However, accounting for additional factors would be unlikely to change our finding that deficits in screening are substantial among both privately and publicly insured children.

## Conclusions

This cross-sectional study found that similar proportions of children diagnosed with obesity at well-child visits received recommended and nonrecommended screening tests for obesity-related co-occurring conditions. The AAP is expected to release a new obesity clinical practice guideline in the second half of 2022. Our findings suggest that these guidelines could be most beneficial if they explicitly recommend a parsimonious set of tests, more emphatically discourage unnecessary testing, and include guidance on implementation strategies, such as clinical decision support tools, which could increase the use of appropriate testing and decrease the use of inappropriate testing. Future studies might monitor changes in screening after the release of updated guidelines.

## Notes

Supplement. **eTable 1.** *ICD-10-CM* Diagnosis Codes for Obesity **eTable 2.** *CPT* Codes for Well-Child Visits **eTable 3.** *CPT* Codes for Laboratory Tests That Could Be Used to Screen for Co-occurring Conditions Among Children With Obesity **eTable 4.** *ICD-10-CM* Diagnosis Codes for Conditions That Potentially Warrant Thyroid and/or Insulin Tests
