# Errors in Data

**DOI:** 10.1001/jamanetworkopen.2022.28489

**Published:** 2022-08-02

The Original Investigation titled “Assessment of Underuse and Overuse of Screening Tests for Co-occurring Conditions Among Children With Obesity,”^1^ published July 14, 2022, has been corrected to fix an error in data that affected the Abstract, Results, and Discussion, as well as Table 2. The number of children who received potentially unnecessary thyroid or insulin tests has been changed to 36 032 (23.0%). This article has been corrected.^1^
