# Supplementary material for: Assessment of Underuse and Overuse of Screening Tests for Co-occurring Conditions Among Children With Obesity
Source: JAMA Netw Open. 2022 Jul 14;5(7):e2222101. doi: 10.1001/jamanetworkopen.2022.22101 (PMC9284328; doi:10.1001/jamanetworkopen.2022.22101)
Supplement: Supplement. — eTable 1. ICD-10-CM Diagnosis Codes for Obesity eTable 2. CPT Codes for Well-Child Visits eTable 3. CPT Codes for Laboratory Tests That Could Be Used to Screen for Co-occurring Conditions Among Children With Obesity eTable 4. ICD-10-CM Diagnosis Codes for Conditions That Potentially Warrant Thyroid and/or Insulin Tests [file jamanetwopen-e2222101-s001.pdf]

Supplementary Online Content

Sharifi M, Goodman AB, Chua KP. Assessment of underuse and overuse of screening tests for co-occurring conditions among children with obesity. *JAMA Netw Open*. 2022;5(7):e2222101. doi:10.1001/jamanetworkopen.2022.22101

eTable 1. *ICD-10-CM* Diagnosis Codes for Obesity

eTable 2. *CPT* Codes for Well-Child Care Visits

eTable 3. *CPT* Codes for Laboratory Tests That Could Be Used to Screen for Co-occurring Conditions Among Children With Obesity

eTable 4. *ICD-10-CM* Diagnosis Codes for Conditions That Potentially Warrant Thyroid and/or Insulin Tests

This supplementary material has been provided by the authors to give readers additional information about their work.

© 2022 Sharifi M et al. *JAMA Network Open*.

eTable 1. *ICD-10-CM* Diagnosis Codes for Obesity

ICD-10-CM diagnosis code	Description
E660	Obesity due to excess calories
E6601	Morbid (severe) obesity due to excess calories
E6609	Other obesity due to excess calories
E661	Drug-induced obesity
E662	Morbid (severe) obesity with alveolar hypoventilation
E668	Other obesity
E669	Obesity, unspecified
Z683	Body mass index (BMI) 30-39, adult
Z6830	Body mass index (BMI) 300-309, adult
Z6831	Body mass index (BMI) 310-319, adult
Z6832	Body mass index (BMI) 320-329, adult
Z6833	Body mass index (BMI) 330-339, adult
Z6834	Body mass index (BMI) 340-349, adult
Z6835	Body mass index (BMI) 350-359, adult
Z6836	Body mass index (BMI) 360-369, adult
Z6837	Body mass index (BMI) 370-379, adult
Z6838	Body mass index (BMI) 380-389, adult
Z6839	Body mass index (BMI) 390-399, adult
Z684	Body mass index (BMI) 40 or greater, adult
Z6841	Body mass index (BMI) 400-449, adult
Z6842	Body mass index (BMI) 450-499, adult
Z6843	Body mass index (BMI) 50-599, adult
Z6844	Body mass index (BMI) 600-699, adult
Z6845	Body mass index (BMI) 70 or greater, adult
Z6854	Body mass index (BMI) pediatric, greater than or equal to 95th percentile for age

© 2022 Sharifi M et al. *JAMA Network Open*.

eTable 2. *CPT* Codes for Well-Child Care Visits

CPT Code	Description
99381	Initial comprehensive preventive medicine evaluation and management of an individual including an age and gender appropriate history, examination, counseling/anticipatory guidance/risk factor reduction interventions, and the ordering of laboratory/diagnostic procedures, new patient; infant (age younger than 1 year)
99382	Initial comprehensive preventive medicine evaluation and management of an individual including an age and gender appropriate history, examination, counseling/anticipatory guidance/risk factor reduction interventions, and the ordering of laboratory/diagnostic procedures, new patient; early childhood (age 1 through 4 years)
99383	Initial comprehensive preventive medicine evaluation and management of an individual including an age and gender appropriate history, examination, counseling/anticipatory guidance/risk factor reduction interventions, and the ordering of laboratory/diagnostic procedures, new patient; late childhood (age 5 through 11 years)
99384	Initial comprehensive preventive medicine evaluation and management of an individual including an age and gender appropriate history, examination, counseling/anticipatory guidance/risk factor reduction interventions, and the ordering of laboratory/diagnostic procedures, new patient; adolescent (age 12 through 17 years)
99385	Initial comprehensive preventive medicine evaluation and management of an individual including an age and gender appropriate history, examination, counseling/anticipatory guidance/risk factor reduction interventions, and the ordering of laboratory/diagnostic procedures, new patient; 18-39 years
99391	Periodic comprehensive preventive medicine reevaluation and management of an individual including an age and gender appropriate history, examination, counseling/anticipatory guidance/risk factor reduction interventions, and the ordering of laboratory/diagnostic procedures, established patient; infant (age younger than 1 year)
99392	Periodic comprehensive preventive medicine reevaluation and management of an individual including an age and gender appropriate history, examination, counseling/anticipatory guidance/risk factor reduction interventions, and the ordering of laboratory/diagnostic procedures, established patient; early childhood (age 1 through 4 years)
99393	Periodic comprehensive preventive medicine reevaluation and management of an individual including an age and gender appropriate history, examination, counseling/anticipatory guidance/risk factor reduction interventions, and the ordering of laboratory/diagnostic procedures, established patient; late childhood (age 5 through 11 years)
99394	Periodic comprehensive preventive medicine reevaluation and management of an individual including an age and gender appropriate history, examination, counseling/anticipatory guidance/risk factor reduction interventions, and the ordering of laboratory/diagnostic procedures, established patient; adolescent (age 12 through 17 years)
99395	Periodic comprehensive preventive medicine reevaluation and management of an individual including an age and gender appropriate history, examination, counseling/anticipatory guidance/risk factor reduction interventions, and the ordering of laboratory/diagnostic procedures, established patient; 18-39 years

© 2022 Sharifi M et al. *JAMA Network Open*.

eTable 3. *CPT* Codes for Laboratory Tests That Could Be Used to Screen for Co-occurring Conditions Among Children With Obesity

CPT code	Description
80047	Basic metabolic panel (Calcium, ionized) This panel must include the following: Calcium, ionized (82330) Carbon dioxide (bicarbonate) (82374) Chloride (82435) Creatinine (82565) Glucose (82947) Potassium (84132) Sodium (84295) Urea Nitrogen (BUN) (84520)
80048	Basic metabolic panel (Calcium, total) This panel must include the following: Calcium, total (82310) Carbon dioxide (bicarbonate) (82374) Chloride (82435) Creatinine (82565) Glucose (82947) Potassium (84132) Sodium (84295) Urea nitrogen (BUN) (84520)
80050	General health panel This panel must include the following: Comprehensive metabolic panel (80053) Blood count, complete (CBC), automated and automated differential WBC count (85025 or 85027 and 85004) OR Blood count, complete (CBC), automated (85027) and appropriate manual differential WBC count (85007 or 85009) Thyroid stimulating hormone (TSH) (84443)
80051	Electrolyte panel This panel must include the following: Carbon dioxide (bicarbonate) (82374) Chloride (82435) Potassium (84132) Sodium (84295)
80053	Comprehensive metabolic panel This panel must include the following: Albumin (82040) Bilirubin, total (82247) Calcium, total (82310) Carbon dioxide (bicarbonate) (82374) Chloride (82435) Creatinine (82565) Glucose (82947) Phosphatase, alkaline (84075) Potassium (84132) Protein, total (84155) Sodium (84295) Transferase, alanine amino (ALT) (SGPT) (84460) Transferase, aspartate amino (AST) (SGOT) (84450) Urea nitrogen (BUN) (84520)
80061	Lipid panel This panel must include the following: Cholesterol, serum, total (82465) Lipoprotein, direct measurement, high density cholesterol (HDL cholesterol) (83718) Triglycerides (84478)
80069	Renal function panel This panel must include the following: Albumin (82040) Calcium, total (82310) Carbon dioxide (bicarbonate) (82374) Chloride (82435) Creatinine (82565) Glucose (82947) Phosphorus inorganic (phosphate) (84100) Potassium (84132) Sodium (84295) Urea nitrogen (BUN) (84520)
80076	Hepatic function panel This panel must include the following: Albumin (82040) Bilirubin, total (82247) Bilirubin, direct (82248) Phosphatase, alkaline (84075) Protein, total (84155) Transferase, alanine amino (ALT) (SGPT) (84460) Transferase, aspartate amino (AST) (SGOT) (84450)
84450	Transferase; aspartate amino (AST) (SGOT)
84460	Transferase; alanine amino (ALT) (SGPT)
84443	Thyroid stimulating hormone (TSH)
82947	Glucose; quantitative, blood (except reagent strip)
82948	Glucose; blood, reagent strip
82950	Glucose; post glucose dose (includes glucose)
82951	Glucose; tolerance test (GTT), 3 specimens (includes glucose)
82952	Glucose; tolerance test, each additional beyond 3 specimens (List separately in addition to code for primary procedure)
83036	Hemoglobin; glycosylated (A1C)
83037	Hemoglobin; glycosylated (A1C) by device cleared by FDA for home use

© 2022 Sharifi M et al. *JAMA Network Open*.

eTable 4. *ICD-10-CM* Diagnosis Codes for Conditions That Potentially Warrant Thyroid and/or Insulin Tests

Code	Description
E00	Congenital iodine-deficiency syndrome
E000	Congenital iodine-deficiency syndrome, neurological type
E001	Congenital iodine-deficiency syndrome, myxedematous type
E002	Congenital iodine-deficiency syndrome, mixed type
E009	Congenital iodine-deficiency syndrome, unspecified
E01	Iodine-deficiency related thyroid disorders and allied conditions
E010	Iodine-deficiency related diffuse (endemic) goiter
E011	Iodine-deficiency related multinodular (endemic) goiter
E012	Iodine-deficiency related (endemic) goiter, unspecified
E018	Other iodine-deficiency related thyroid disorders and allied conditions
E02	Subclinical iodine-deficiency hypothyroidism
E03	Other hypothyroidism
E030	Congenital hypothyroidism with diffuse goiter
E031	Congenital hypothyroidism without goiter
E032	Hypothyroidism due to medicaments and other exogenous substances
E033	Postinfectious hypothyroidism
E034	Atrophy of thyroid (acquired)
E035	Myxedema coma
E038	Other specified hypothyroidism
E039	Hypothyroidism, unspecified
E04	Other nontoxic goiter
E040	Nontoxic diffuse goiter
E041	Nontoxic single thyroid nodule
E042	Nontoxic multinodular goiter
E048	Other specified nontoxic goiter
E049	Nontoxic goiter, unspecified
E05	Thyrotoxicosis [hyperthyroidism]
E050	Thyrotoxicosis with diffuse goiter
E0500	Thyrotoxicosis with diffuse goiter without thyrotoxic crisis or storm
E0501	Thyrotoxicosis with diffuse goiter with thyrotoxic crisis or storm
E051	Thyrotoxicosis with toxic single thyroid nodule
E0510	Thyrotoxicosis with toxic single thyroid nodule without thyrotoxic crisis or storm
E0511	Thyrotoxicosis with toxic single thyroid nodule with thyrotoxic crisis or storm
E052	Thyrotoxicosis with toxic multinodular goiter
E0520	Thyrotoxicosis with toxic multinodular goiter without thyrotoxic crisis or storm
E0521	Thyrotoxicosis with toxic multinodular goiter with thyrotoxic crisis or storm
E053	Thyrotoxicosis from ectopic thyroid tissue
E0530	Thyrotoxicosis from ectopic thyroid tissue without thyrotoxic crisis or storm
E0531	Thyrotoxicosis from ectopic thyroid tissue with thyrotoxic crisis or storm
E054	Thyrotoxicosis factitia
E0540	Thyrotoxicosis factitia without thyrotoxic crisis or storm
E0541	Thyrotoxicosis factitia with thyrotoxic crisis or storm
E058	Other thyrotoxicosis
E0580	Other thyrotoxicosis without thyrotoxic crisis or storm
E0581	Other thyrotoxicosis with thyrotoxic crisis or storm
E059	Thyrotoxicosis, unspecified
E0590	Thyrotoxicosis, unspecified without thyrotoxic crisis or storm
E0591	Thyrotoxicosis, unspecified with thyrotoxic crisis or storm
E06	Thyroiditis
E060	Acute thyroiditis
E061	Subacute thyroiditis
E062	Chronic thyroiditis with transient thyrotoxicosis
E063	Autoimmune thyroiditis

© 2022 Sharifi M et al. *JAMA Network Open*.

E064	Drug-induced thyroiditis
E065	Other chronic thyroiditis
E069	Thyroiditis, unspecified
E07	Other disorders of thyroid
E070	Hypersecretion of calcitonin
E071	Dyshormogenetic goiter
E078	Other specified disorders of thyroid
E0781	Sick-euthyroid syndrome
E0789	Other specified disorders of thyroid
E079	Disorder of thyroid, unspecified
E08	Diabetes mellitus due to underlying condition
E080	Diabetes mellitus due to underlying condition with hyperosmolarity
E0800	Diabetes mellitus due to underlying condition with hyperosmolarity without nonketotic hyperglycemic-hyperosmolar coma (NKHHC)
E0801	Diabetes mellitus due to underlying condition with hyperosmolarity with coma
E081	Diabetes mellitus due to underlying condition with ketoacidosis
E0810	Diabetes mellitus due to underlying condition with ketoacidosis without coma
E0811	Diabetes mellitus due to underlying condition with ketoacidosis with coma
E082	Diabetes mellitus due to underlying condition with kidney complications
E0821	Diabetes mellitus due to underlying condition with diabetic nephropathy
E0822	Diabetes mellitus due to underlying condition with diabetic chronic kidney disease
E0829	Diabetes mellitus due to underlying condition with other diabetic kidney complication
E083	Diabetes mellitus due to underlying condition with ophthalmic complications
E0831	Diabetes mellitus due to underlying condition with unspecified diabetic retinopathy
E08311	Diabetes mellitus due to underlying condition with unspecified diabetic retinopathy with macular edema
E08319	Diabetes mellitus due to underlying condition with unspecified diabetic retinopathy without macular edema
E0832	Diabetes mellitus due to underlying condition with mild nonproliferative diabetic retinopathy
E08321	Diabetes mellitus due to underlying condition with mild nonproliferative diabetic retinopathy with macular edema
E083211	Diabetes mellitus due to underlying condition with mild nonproliferative diabetic retinopathy with macular edema, right eye
E083212	Diabetes mellitus due to underlying condition with mild nonproliferative diabetic retinopathy with macular edema, left eye
E083213	Diabetes mellitus due to underlying condition with mild nonproliferative diabetic retinopathy with macular edema, bilateral
E083219	Diabetes mellitus due to underlying condition with mild nonproliferative diabetic retinopathy with macular edema, unspecified eye
E08329	Diabetes mellitus due to underlying condition with mild nonproliferative diabetic retinopathy without macular edema
E083291	Diabetes mellitus due to underlying condition with mild nonproliferative diabetic retinopathy without macular edema, right eye
E083292	Diabetes mellitus due to underlying condition with mild nonproliferative diabetic retinopathy without macular edema, left eye
E083293	Diabetes mellitus due to underlying condition with mild nonproliferative diabetic retinopathy without macular edema, bilateral
E083299	Diabetes mellitus due to underlying condition with mild nonproliferative diabetic retinopathy without macular edema, unspecified eye
E0833	Diabetes mellitus due to underlying condition with moderate nonproliferative diabetic retinopathy
E08331	Diabetes mellitus due to underlying condition with moderate nonproliferative diabetic retinopathy with macular edema
E083311	Diabetes mellitus due to underlying condition with moderate nonproliferative diabetic retinopathy with macular edema, right eye
E083312	Diabetes mellitus due to underlying condition with moderate nonproliferative diabetic retinopathy with macular edema, left eye
E083313	Diabetes mellitus due to underlying condition with moderate nonproliferative diabetic retinopathy with macular edema, bilateral
E083319	Diabetes mellitus due to underlying condition with moderate nonproliferative diabetic retinopathy with macular edema, unspecified eye

© 2022 Sharifi M et al. *JAMA Network Open*.

E08339	Diabetes mellitus due to underlying condition with moderate nonproliferative diabetic retinopathy without macular edema
E083391	Diabetes mellitus due to underlying condition with moderate nonproliferative diabetic retinopathy without macular edema, right eye
E083392	Diabetes mellitus due to underlying condition with moderate nonproliferative diabetic retinopathy without macular edema, left eye
E083393	Diabetes mellitus due to underlying condition with moderate nonproliferative diabetic retinopathy without macular edema, bilateral
E083399	Diabetes mellitus due to underlying condition with moderate nonproliferative diabetic retinopathy without macular edema, unspecified eye
E0834	Diabetes mellitus due to underlying condition with severe nonproliferative diabetic retinopathy
E08341	Diabetes mellitus due to underlying condition with severe nonproliferative diabetic retinopathy with macular edema
E083411	Diabetes mellitus due to underlying condition with severe nonproliferative diabetic retinopathy with macular edema, right eye
E083412	Diabetes mellitus due to underlying condition with severe nonproliferative diabetic retinopathy with macular edema, left eye
E083413	Diabetes mellitus due to underlying condition with severe nonproliferative diabetic retinopathy with macular edema, bilateral
E083419	Diabetes mellitus due to underlying condition with severe nonproliferative diabetic retinopathy with macular edema, unspecified eye
E08349	Diabetes mellitus due to underlying condition with severe nonproliferative diabetic retinopathy without macular edema
E083491	Diabetes mellitus due to underlying condition with severe nonproliferative diabetic retinopathy without macular edema, right eye
E083492	Diabetes mellitus due to underlying condition with severe nonproliferative diabetic retinopathy without macular edema, left eye
E083493	Diabetes mellitus due to underlying condition with severe nonproliferative diabetic retinopathy without macular edema, bilateral
E083499	Diabetes mellitus due to underlying condition with severe nonproliferative diabetic retinopathy without macular edema, unspecified eye
E0835	Diabetes mellitus due to underlying condition with proliferative diabetic retinopathy
E08351	Diabetes mellitus due to underlying condition with proliferative diabetic retinopathy with macular edema
E083511	Diabetes mellitus due to underlying condition with proliferative diabetic retinopathy with macular edema, right eye
E083512	Diabetes mellitus due to underlying condition with proliferative diabetic retinopathy with macular edema, left eye
E083513	Diabetes mellitus due to underlying condition with proliferative diabetic retinopathy with macular edema, bilateral
E083519	Diabetes mellitus due to underlying condition with proliferative diabetic retinopathy with macular edema, unspecified eye
E08352	Diabetes mellitus due to underlying condition with proliferative diabetic retinopathy with traction retinal detachment involving the macula
E083521	Diabetes mellitus due to underlying condition with proliferative diabetic retinopathy with traction retinal detachment involving the macula, right eye
E083522	Diabetes mellitus due to underlying condition with proliferative diabetic retinopathy with traction retinal detachment involving the macula, left eye
E083523	Diabetes mellitus due to underlying condition with proliferative diabetic retinopathy with traction retinal detachment involving the macula, bilateral
E083529	Diabetes mellitus due to underlying condition with proliferative diabetic retinopathy with traction retinal detachment involving the macula, unspecified eye
E08353	Diabetes mellitus due to underlying condition with proliferative diabetic retinopathy with traction retinal detachment not involving the macula
E083531	Diabetes mellitus due to underlying condition with proliferative diabetic retinopathy with traction retinal detachment not involving the macula, right eye
E083532	Diabetes mellitus due to underlying condition with proliferative diabetic retinopathy with traction retinal detachment not involving the macula, left eye

© 2022 Sharifi M et al. *JAMA Network Open*.

E083533	Diabetes mellitus due to underlying condition with proliferative diabetic retinopathy with traction retinal detachment not involving the macula, bilateral
E083539	Diabetes mellitus due to underlying condition with proliferative diabetic retinopathy with traction retinal detachment not involving the macula, unspecified eye
E08354	Diabetes mellitus due to underlying condition with proliferative diabetic retinopathy with combined traction retinal detachment and rhegmatogenous retinal detachment
E083541	Diabetes mellitus due to underlying condition with proliferative diabetic retinopathy with combined traction retinal detachment and rhegmatogenous retinal detachment, right eye
E083542	Diabetes mellitus due to underlying condition with proliferative diabetic retinopathy with combined traction retinal detachment and rhegmatogenous retinal detachment, left eye
E083543	Diabetes mellitus due to underlying condition with proliferative diabetic retinopathy with combined traction retinal detachment and rhegmatogenous retinal detachment, bilateral
E083549	Diabetes mellitus due to underlying condition with proliferative diabetic retinopathy with combined traction retinal detachment and rhegmatogenous retinal detachment, unspecified eye
E08355	Diabetes mellitus due to underlying condition with stable proliferative diabetic retinopathy
E083551	Diabetes mellitus due to underlying condition with stable proliferative diabetic retinopathy, right eye
E083552	Diabetes mellitus due to underlying condition with stable proliferative diabetic retinopathy, left eye
E083553	Diabetes mellitus due to underlying condition with stable proliferative diabetic retinopathy, bilateral
E083559	Diabetes mellitus due to underlying condition with stable proliferative diabetic retinopathy, unspecified eye
E08359	Diabetes mellitus due to underlying condition with proliferative diabetic retinopathy without macular edema
E083591	Diabetes mellitus due to underlying condition with proliferative diabetic retinopathy without macular edema, right eye
E083592	Diabetes mellitus due to underlying condition with proliferative diabetic retinopathy without macular edema, left eye
E083593	Diabetes mellitus due to underlying condition with proliferative diabetic retinopathy without macular edema, bilateral
E083599	Diabetes mellitus due to underlying condition with proliferative diabetic retinopathy without macular edema, unspecified eye
E0836	Diabetes mellitus due to underlying condition with diabetic cataract
E0837	Diabetes mellitus due to underlying condition with diabetic macular edema, resolved following treatment
E0837X1	Diabetes mellitus due to underlying condition with diabetic macular edema, resolved following treatment, right eye
E0837X2	Diabetes mellitus due to underlying condition with diabetic macular edema, resolved following treatment, left eye
E0837X3	Diabetes mellitus due to underlying condition with diabetic macular edema, resolved following treatment, bilateral
E0837X9	Diabetes mellitus due to underlying condition with diabetic macular edema, resolved following treatment, unspecified eye
E0839	Diabetes mellitus due to underlying condition with other diabetic ophthalmic complication
E084	Diabetes mellitus due to underlying condition with neurological complications
E0840	Diabetes mellitus due to underlying condition with diabetic neuropathy, unspecified
E0841	Diabetes mellitus due to underlying condition with diabetic mononeuropathy
E0842	Diabetes mellitus due to underlying condition with diabetic polyneuropathy
E0843	Diabetes mellitus due to underlying condition with diabetic autonomic (poly)neuropathy
E0844	Diabetes mellitus due to underlying condition with diabetic amyotrophy
E0849	Diabetes mellitus due to underlying condition with other diabetic neurological complication
E085	Diabetes mellitus due to underlying condition with circulatory complications
E0851	Diabetes mellitus due to underlying condition with diabetic peripheral angiopathy without gangrene
E0852	Diabetes mellitus due to underlying condition with diabetic peripheral angiopathy with gangrene
E0859	Diabetes mellitus due to underlying condition with other circulatory complications
E086	Diabetes mellitus due to underlying condition with other specified complications
E0861	Diabetes mellitus due to underlying condition with diabetic arthropathy
E08610	Diabetes mellitus due to underlying condition with diabetic neuropathic arthropathy
E08618	Diabetes mellitus due to underlying condition with other diabetic arthropathy
E0862	Diabetes mellitus due to underlying condition with skin complications
E08620	Diabetes mellitus due to underlying condition with diabetic dermatitis
E08621	Diabetes mellitus due to underlying condition with foot ulcer
E08622	Diabetes mellitus due to underlying condition with other skin ulcer

© 2022 Sharifi M et al. *JAMA Network Open*.

E08628	Diabetes mellitus due to underlying condition with other skin complications
E0863	Diabetes mellitus due to underlying condition with oral complications
E08630	Diabetes mellitus due to underlying condition with periodontal disease
E08638	Diabetes mellitus due to underlying condition with other oral complications
E0864	Diabetes mellitus due to underlying condition with hypoglycemia
E08641	Diabetes mellitus due to underlying condition with hypoglycemia with coma
E08649	Diabetes mellitus due to underlying condition with hypoglycemia without coma
E0865	Diabetes mellitus due to underlying condition with hyperglycemia
E0869	Diabetes mellitus due to underlying condition with other specified complication
E088	Diabetes mellitus due to underlying condition with unspecified complications
E089	Diabetes mellitus due to underlying condition without complications
E09	Drug or chemical induced diabetes mellitus
E090	Drug or chemical induced diabetes mellitus with hyperosmolarity
E0900	Drug or chemical induced diabetes mellitus with hyperosmolarity without nonketotic hyperglycemic-hyperosmolar coma (NKHHC)
E0901	Drug or chemical induced diabetes mellitus with hyperosmolarity with coma
E091	Drug or chemical induced diabetes mellitus with ketoacidosis
E0910	Drug or chemical induced diabetes mellitus with ketoacidosis without coma
E0911	Drug or chemical induced diabetes mellitus with ketoacidosis with coma
E092	Drug or chemical induced diabetes mellitus with kidney complications
E0921	Drug or chemical induced diabetes mellitus with diabetic nephropathy
E0922	Drug or chemical induced diabetes mellitus with diabetic chronic kidney disease
E0929	Drug or chemical induced diabetes mellitus with other diabetic kidney complication
E093	Drug or chemical induced diabetes mellitus with ophthalmic complications
E0931	Drug or chemical induced diabetes mellitus with unspecified diabetic retinopathy
E09311	Drug or chemical induced diabetes mellitus with unspecified diabetic retinopathy with macular edema
E09319	Drug or chemical induced diabetes mellitus with unspecified diabetic retinopathy without macular edema
E0932	Drug or chemical induced diabetes mellitus with mild nonproliferative diabetic retinopathy
E09321	Drug or chemical induced diabetes mellitus with mild nonproliferative diabetic retinopathy with macular edema
E093211	Drug or chemical induced diabetes mellitus with mild nonproliferative diabetic retinopathy with macular edema, right eye
E093212	Drug or chemical induced diabetes mellitus with mild nonproliferative diabetic retinopathy with macular edema, left eye
E093213	Drug or chemical induced diabetes mellitus with mild nonproliferative diabetic retinopathy with macular edema, bilateral
E093219	Drug or chemical induced diabetes mellitus with mild nonproliferative diabetic retinopathy with macular edema, unspecified eye
E09329	Drug or chemical induced diabetes mellitus with mild nonproliferative diabetic retinopathy without macular edema
E093291	Drug or chemical induced diabetes mellitus with mild nonproliferative diabetic retinopathy without macular edema, right eye
E093292	Drug or chemical induced diabetes mellitus with mild nonproliferative diabetic retinopathy without macular edema, left eye
E093293	Drug or chemical induced diabetes mellitus with mild nonproliferative diabetic retinopathy without macular edema, bilateral
E093299	Drug or chemical induced diabetes mellitus with mild nonproliferative diabetic retinopathy without macular edema, unspecified eye
E0933	Drug or chemical induced diabetes mellitus with moderate nonproliferative diabetic retinopathy
E09331	Drug or chemical induced diabetes mellitus with moderate nonproliferative diabetic retinopathy with macular edema
E093311	Drug or chemical induced diabetes mellitus with moderate nonproliferative diabetic retinopathy with macular edema, right eye
E093312	Drug or chemical induced diabetes mellitus with moderate nonproliferative diabetic retinopathy with macular edema, left eye
E093313	Drug or chemical induced diabetes mellitus with moderate nonproliferative diabetic retinopathy with macular edema, bilateral

© 2022 Sharifi M et al. *JAMA Network Open*.

E093319	Drug or chemical induced diabetes mellitus with moderate nonproliferative diabetic retinopathy with macular edema, unspecified eye
E09339	Drug or chemical induced diabetes mellitus with moderate nonproliferative diabetic retinopathy without macular edema
E093391	Drug or chemical induced diabetes mellitus with moderate nonproliferative diabetic retinopathy without macular edema, right eye
E093392	Drug or chemical induced diabetes mellitus with moderate nonproliferative diabetic retinopathy without macular edema, left eye
E093393	Drug or chemical induced diabetes mellitus with moderate nonproliferative diabetic retinopathy without macular edema, bilateral
E093399	Drug or chemical induced diabetes mellitus with moderate nonproliferative diabetic retinopathy without macular edema, unspecified eye
E0934	Drug or chemical induced diabetes mellitus with severe nonproliferative diabetic retinopathy
E09341	Drug or chemical induced diabetes mellitus with severe nonproliferative diabetic retinopathy with macular edema
E093411	Drug or chemical induced diabetes mellitus with severe nonproliferative diabetic retinopathy with macular edema, right eye
E093412	Drug or chemical induced diabetes mellitus with severe nonproliferative diabetic retinopathy with macular edema, left eye
E093413	Drug or chemical induced diabetes mellitus with severe nonproliferative diabetic retinopathy with macular edema, bilateral
E093419	Drug or chemical induced diabetes mellitus with severe nonproliferative diabetic retinopathy with macular edema, unspecified eye
E09349	Drug or chemical induced diabetes mellitus with severe nonproliferative diabetic retinopathy without macular edema
E093491	Drug or chemical induced diabetes mellitus with severe nonproliferative diabetic retinopathy without macular edema, right eye
E093492	Drug or chemical induced diabetes mellitus with severe nonproliferative diabetic retinopathy without macular edema, left eye
E093493	Drug or chemical induced diabetes mellitus with severe nonproliferative diabetic retinopathy without macular edema, bilateral
E093499	Drug or chemical induced diabetes mellitus with severe nonproliferative diabetic retinopathy without macular edema, unspecified eye
E0935	Drug or chemical induced diabetes mellitus with proliferative diabetic retinopathy
E09351	Drug or chemical induced diabetes mellitus with proliferative diabetic retinopathy with macular edema
E093511	Drug or chemical induced diabetes mellitus with proliferative diabetic retinopathy with macular edema, right eye
E093512	Drug or chemical induced diabetes mellitus with proliferative diabetic retinopathy with macular edema, left eye
E093513	Drug or chemical induced diabetes mellitus with proliferative diabetic retinopathy with macular edema, bilateral
E093519	Drug or chemical induced diabetes mellitus with proliferative diabetic retinopathy with macular edema, unspecified eye
E09352	Drug or chemical induced diabetes mellitus with proliferative diabetic retinopathy with traction retinal detachment involving the macula
E093521	Drug or chemical induced diabetes mellitus with proliferative diabetic retinopathy with traction retinal detachment involving the macula, right eye
E093522	Drug or chemical induced diabetes mellitus with proliferative diabetic retinopathy with traction retinal detachment involving the macula, left eye
E093523	Drug or chemical induced diabetes mellitus with proliferative diabetic retinopathy with traction retinal detachment involving the macula, bilateral
E093529	Drug or chemical induced diabetes mellitus with proliferative diabetic retinopathy with traction retinal detachment involving the macula, unspecified eye
E09353	Drug or chemical induced diabetes mellitus with proliferative diabetic retinopathy with traction retinal detachment not involving the macula
E093531	Drug or chemical induced diabetes mellitus with proliferative diabetic retinopathy with traction retinal detachment not involving the macula, right eye

© 2022 Sharifi M et al. *JAMA Network Open*.

E093532	Drug or chemical induced diabetes mellitus with proliferative diabetic retinopathy with traction retinal detachment not involving the macula, left eye
E093533	Drug or chemical induced diabetes mellitus with proliferative diabetic retinopathy with traction retinal detachment not involving the macula, bilateral
E093539	Drug or chemical induced diabetes mellitus with proliferative diabetic retinopathy with traction retinal detachment not involving the macula, unspecified eye
E09354	Drug or chemical induced diabetes mellitus with proliferative diabetic retinopathy with combined traction retinal detachment and rhegmatogenous retinal detachment
E093541	Drug or chemical induced diabetes mellitus with proliferative diabetic retinopathy with combined traction retinal detachment and rhegmatogenous retinal detachment, right eye
E093542	Drug or chemical induced diabetes mellitus with proliferative diabetic retinopathy with combined traction retinal detachment and rhegmatogenous retinal detachment, left eye
E093543	Drug or chemical induced diabetes mellitus with proliferative diabetic retinopathy with combined traction retinal detachment and rhegmatogenous retinal detachment, bilateral
E093549	Drug or chemical induced diabetes mellitus with proliferative diabetic retinopathy with combined traction retinal detachment and rhegmatogenous retinal detachment, unspecified eye
E09355	Drug or chemical induced diabetes mellitus with stable proliferative diabetic retinopathy
E093551	Drug or chemical induced diabetes mellitus with stable proliferative diabetic retinopathy, right eye
E093552	Drug or chemical induced diabetes mellitus with stable proliferative diabetic retinopathy, left eye
E093553	Drug or chemical induced diabetes mellitus with stable proliferative diabetic retinopathy, bilateral
E093559	Drug or chemical induced diabetes mellitus with stable proliferative diabetic retinopathy, unspecified eye
E09359	Drug or chemical induced diabetes mellitus with proliferative diabetic retinopathy without macular edema
E093591	Drug or chemical induced diabetes mellitus with proliferative diabetic retinopathy without macular edema, right eye
E093592	Drug or chemical induced diabetes mellitus with proliferative diabetic retinopathy without macular edema, left eye
E093593	Drug or chemical induced diabetes mellitus with proliferative diabetic retinopathy without macular edema, bilateral
E093599	Drug or chemical induced diabetes mellitus with proliferative diabetic retinopathy without macular edema, unspecified eye
E0936	Drug or chemical induced diabetes mellitus with diabetic cataract
E0937	Drug or chemical induced diabetes mellitus with diabetic macular edema, resolved following treatment
E0937X1	Drug or chemical induced diabetes mellitus with diabetic macular edema, resolved following treatment, right eye
E0937X2	Drug or chemical induced diabetes mellitus with diabetic macular edema, resolved following treatment, left eye
E0937X3	Drug or chemical induced diabetes mellitus with diabetic macular edema, resolved following treatment, bilateral
E0937X9	Drug or chemical induced diabetes mellitus with diabetic macular edema, resolved following treatment, unspecified eye
E0939	Drug or chemical induced diabetes mellitus with other diabetic ophthalmic complication
E094	Drug or chemical induced diabetes mellitus with neurological complications
E0940	Drug or chemical induced diabetes mellitus with neurological complications with diabetic neuropathy, unspecified
E0941	Drug or chemical induced diabetes mellitus with neurological complications with diabetic mononeuropathy
E0942	Drug or chemical induced diabetes mellitus with neurological complications with diabetic polyneuropathy
E0943	Drug or chemical induced diabetes mellitus with neurological complications with diabetic autonomic (poly)neuropathy
E0944	Drug or chemical induced diabetes mellitus with neurological complications with diabetic amyotrophy
E0949	Drug or chemical induced diabetes mellitus with neurological complications with other diabetic neurological complication
E095	Drug or chemical induced diabetes mellitus with circulatory complications
E0951	Drug or chemical induced diabetes mellitus with diabetic peripheral angiopathy without gangrene
E0952	Drug or chemical induced diabetes mellitus with diabetic peripheral angiopathy with gangrene
E0959	Drug or chemical induced diabetes mellitus with other circulatory complications
E096	Drug or chemical induced diabetes mellitus with other specified complications
E0961	Drug or chemical induced diabetes mellitus with diabetic arthropathy
E09610	Drug or chemical induced diabetes mellitus with diabetic neuropathic arthropathy

© 2022 Sharifi M et al. *JAMA Network Open*.

E09618	Drug or chemical induced diabetes mellitus with other diabetic arthropathy
E0962	Drug or chemical induced diabetes mellitus with skin complications
E09620	Drug or chemical induced diabetes mellitus with diabetic dermatitis
E09621	Drug or chemical induced diabetes mellitus with foot ulcer
E09622	Drug or chemical induced diabetes mellitus with other skin ulcer
E09628	Drug or chemical induced diabetes mellitus with other skin complications
E0963	Drug or chemical induced diabetes mellitus with oral complications
E09630	Drug or chemical induced diabetes mellitus with periodontal disease
E09638	Drug or chemical induced diabetes mellitus with other oral complications
E0964	Drug or chemical induced diabetes mellitus with hypoglycemia
E09641	Drug or chemical induced diabetes mellitus with hypoglycemia with coma
E09649	Drug or chemical induced diabetes mellitus with hypoglycemia without coma
E0965	Drug or chemical induced diabetes mellitus with hyperglycemia
E0969	Drug or chemical induced diabetes mellitus with other specified complication
E098	Drug or chemical induced diabetes mellitus with unspecified complications
E099	Drug or chemical induced diabetes mellitus without complications
E10	Type 1 diabetes mellitus
E101	Type 1 diabetes mellitus with ketoacidosis
E1010	Type 1 diabetes mellitus with ketoacidosis without coma
E1011	Type 1 diabetes mellitus with ketoacidosis with coma
E102	Type 1 diabetes mellitus with kidney complications
E1021	Type 1 diabetes mellitus with diabetic nephropathy
E1022	Type 1 diabetes mellitus with diabetic chronic kidney disease
E1029	Type 1 diabetes mellitus with other diabetic kidney complication
E103	Type 1 diabetes mellitus with ophthalmic complications
E1031	Type 1 diabetes mellitus with unspecified diabetic retinopathy
E10311	Type 1 diabetes mellitus with unspecified diabetic retinopathy with macular edema
E10319	Type 1 diabetes mellitus with unspecified diabetic retinopathy without macular edema
E1032	Type 1 diabetes mellitus with mild nonproliferative diabetic retinopathy
E10321	Type 1 diabetes mellitus with mild nonproliferative diabetic retinopathy with macular edema
E103211	Type 1 diabetes mellitus with mild nonproliferative diabetic retinopathy with macular edema, right eye
E103212	Type 1 diabetes mellitus with mild nonproliferative diabetic retinopathy with macular edema, left eye
E103213	Type 1 diabetes mellitus with mild nonproliferative diabetic retinopathy with macular edema, bilateral
E103219	Type 1 diabetes mellitus with mild nonproliferative diabetic retinopathy with macular edema, unspecified eye
E10329	Type 1 diabetes mellitus with mild nonproliferative diabetic retinopathy without macular edema
E103291	Type 1 diabetes mellitus with mild nonproliferative diabetic retinopathy without macular edema, right eye
E103292	Type 1 diabetes mellitus with mild nonproliferative diabetic retinopathy without macular edema, left eye
E103293	Type 1 diabetes mellitus with mild nonproliferative diabetic retinopathy without macular edema, bilateral
E103299	Type 1 diabetes mellitus with mild nonproliferative diabetic retinopathy without macular edema, unspecified eye
E1033	Type 1 diabetes mellitus with moderate nonproliferative diabetic retinopathy
E10331	Type 1 diabetes mellitus with moderate nonproliferative diabetic retinopathy with macular edema
E103311	Type 1 diabetes mellitus with moderate nonproliferative diabetic retinopathy with macular edema, right eye
E103312	Type 1 diabetes mellitus with moderate nonproliferative diabetic retinopathy with macular edema, left eye
E103313	Type 1 diabetes mellitus with moderate nonproliferative diabetic retinopathy with macular edema, bilateral
E103319	Type 1 diabetes mellitus with moderate nonproliferative diabetic retinopathy with macular edema, unspecified eye
E10339	Type 1 diabetes mellitus with moderate nonproliferative diabetic retinopathy without macular edema
E103391	Type 1 diabetes mellitus with moderate nonproliferative diabetic retinopathy without macular edema, right eye
E103392	Type 1 diabetes mellitus with moderate nonproliferative diabetic retinopathy without macular edema, left eye
E103393	Type 1 diabetes mellitus with moderate nonproliferative diabetic retinopathy without macular edema, bilateral
E103399	Type 1 diabetes mellitus with moderate nonproliferative diabetic retinopathy without macular edema, unspecified eye

© 2022 Sharifi M et al. *JAMA Network Open*.

E1034	Type 1 diabetes mellitus with severe nonproliferative diabetic retinopathy
E10341	Type 1 diabetes mellitus with severe nonproliferative diabetic retinopathy with macular edema
E103411	Type 1 diabetes mellitus with severe nonproliferative diabetic retinopathy with macular edema, right eye
E103412	Type 1 diabetes mellitus with severe nonproliferative diabetic retinopathy with macular edema, left eye
E103413	Type 1 diabetes mellitus with severe nonproliferative diabetic retinopathy with macular edema, bilateral
E103419	Type 1 diabetes mellitus with severe nonproliferative diabetic retinopathy with macular edema, unspecified eye
E10349	Type 1 diabetes mellitus with severe nonproliferative diabetic retinopathy without macular edema
E103491	Type 1 diabetes mellitus with severe nonproliferative diabetic retinopathy without macular edema, right eye
E103492	Type 1 diabetes mellitus with severe nonproliferative diabetic retinopathy without macular edema, left eye
E103493	Type 1 diabetes mellitus with severe nonproliferative diabetic retinopathy without macular edema, bilateral
E103499	Type 1 diabetes mellitus with severe nonproliferative diabetic retinopathy without macular edema, unspecified eye
E1035	Type 1 diabetes mellitus with proliferative diabetic retinopathy
E10351	Type 1 diabetes mellitus with proliferative diabetic retinopathy with macular edema
E103511	Type 1 diabetes mellitus with proliferative diabetic retinopathy with macular edema, right eye
E103512	Type 1 diabetes mellitus with proliferative diabetic retinopathy with macular edema, left eye
E103513	Type 1 diabetes mellitus with proliferative diabetic retinopathy with macular edema, bilateral
E103519	Type 1 diabetes mellitus with proliferative diabetic retinopathy with macular edema, unspecified eye
E10352	Type 1 diabetes mellitus with proliferative diabetic retinopathy with traction retinal detachment involving the macula
E103521	Type 1 diabetes mellitus with proliferative diabetic retinopathy with traction retinal detachment involving the macula, right eye
E103522	Type 1 diabetes mellitus with proliferative diabetic retinopathy with traction retinal detachment involving the macula, left eye
E103523	Type 1 diabetes mellitus with proliferative diabetic retinopathy with traction retinal detachment involving the macula, bilateral
E103529	Type 1 diabetes mellitus with proliferative diabetic retinopathy with traction retinal detachment involving the macula, unspecified eye
E10353	Type 1 diabetes mellitus with proliferative diabetic retinopathy with traction retinal detachment not involving the macula
E103531	Type 1 diabetes mellitus with proliferative diabetic retinopathy with traction retinal detachment not involving the macula, right eye
E103532	Type 1 diabetes mellitus with proliferative diabetic retinopathy with traction retinal detachment not involving the macula, left eye
E103533	Type 1 diabetes mellitus with proliferative diabetic retinopathy with traction retinal detachment not involving the macula, bilateral
E103539	Type 1 diabetes mellitus with proliferative diabetic retinopathy with traction retinal detachment not involving the macula, unspecified eye
E10354	Type 1 diabetes mellitus with proliferative diabetic retinopathy with combined traction retinal detachment and rhegmatogenous retinal detachment
E103541	Type 1 diabetes mellitus with proliferative diabetic retinopathy with combined traction retinal detachment and rhegmatogenous retinal detachment, right eye
E103542	Type 1 diabetes mellitus with proliferative diabetic retinopathy with combined traction retinal detachment and rhegmatogenous retinal detachment, left eye
E103543	Type 1 diabetes mellitus with proliferative diabetic retinopathy with combined traction retinal detachment and rhegmatogenous retinal detachment, bilateral
E103549	Type 1 diabetes mellitus with proliferative diabetic retinopathy with combined traction retinal detachment and rhegmatogenous retinal detachment, unspecified eye
E10355	Type 1 diabetes mellitus with stable proliferative diabetic retinopathy
E103551	Type 1 diabetes mellitus with stable proliferative diabetic retinopathy, right eye
E103552	Type 1 diabetes mellitus with stable proliferative diabetic retinopathy, left eye
E103553	Type 1 diabetes mellitus with stable proliferative diabetic retinopathy, bilateral
E103559	Type 1 diabetes mellitus with stable proliferative diabetic retinopathy, unspecified eye
E10359	Type 1 diabetes mellitus with proliferative diabetic retinopathy without macular edema
E103591	Type 1 diabetes mellitus with proliferative diabetic retinopathy without macular edema, right eye
E103592	Type 1 diabetes mellitus with proliferative diabetic retinopathy without macular edema, left eye
E103593	Type 1 diabetes mellitus with proliferative diabetic retinopathy without macular edema, bilateral

© 2022 Sharifi M et al. *JAMA Network Open*.

E103599	Type 1 diabetes mellitus with proliferative diabetic retinopathy without macular edema, unspecified eye
E1036	Type 1 diabetes mellitus with diabetic cataract
E1037	Type 1 diabetes mellitus with diabetic macular edema, resolved following treatment
E1037X1	Type 1 diabetes mellitus with diabetic macular edema, resolved following treatment, right eye
E1037X2	Type 1 diabetes mellitus with diabetic macular edema, resolved following treatment, left eye
E1037X3	Type 1 diabetes mellitus with diabetic macular edema, resolved following treatment, bilateral
E1037X9	Type 1 diabetes mellitus with diabetic macular edema, resolved following treatment, unspecified eye
E1039	Type 1 diabetes mellitus with other diabetic ophthalmic complication
E104	Type 1 diabetes mellitus with neurological complications
E1040	Type 1 diabetes mellitus with diabetic neuropathy, unspecified
E1041	Type 1 diabetes mellitus with diabetic mononeuropathy
E1042	Type 1 diabetes mellitus with diabetic polyneuropathy
E1043	Type 1 diabetes mellitus with diabetic autonomic (poly)neuropathy
E1044	Type 1 diabetes mellitus with diabetic amyotrophy
E1049	Type 1 diabetes mellitus with other diabetic neurological complication
E105	Type 1 diabetes mellitus with circulatory complications
E1051	Type 1 diabetes mellitus with diabetic peripheral angiopathy without gangrene
E1052	Type 1 diabetes mellitus with diabetic peripheral angiopathy with gangrene
E1059	Type 1 diabetes mellitus with other circulatory complications
E106	Type 1 diabetes mellitus with other specified complications
E1061	Type 1 diabetes mellitus with diabetic arthropathy
E10610	Type 1 diabetes mellitus with diabetic neuropathic arthropathy
E10618	Type 1 diabetes mellitus with other diabetic arthropathy
E1062	Type 1 diabetes mellitus with skin complications
E10620	Type 1 diabetes mellitus with diabetic dermatitis
E10621	Type 1 diabetes mellitus with foot ulcer
E10622	Type 1 diabetes mellitus with other skin ulcer
E10628	Type 1 diabetes mellitus with other skin complications
E1063	Type 1 diabetes mellitus with oral complications
E10630	Type 1 diabetes mellitus with periodontal disease
E10638	Type 1 diabetes mellitus with other oral complications
E1064	Type 1 diabetes mellitus with hypoglycemia
E10641	Type 1 diabetes mellitus with hypoglycemia with coma
E10649	Type 1 diabetes mellitus with hypoglycemia without coma
E1065	Type 1 diabetes mellitus with hyperglycemia
E1069	Type 1 diabetes mellitus with other specified complication
E108	Type 1 diabetes mellitus with unspecified complications
E109	Type 1 diabetes mellitus without complications
E11	Type 2 diabetes mellitus
E110	Type 2 diabetes mellitus with hyperosmolarity
E1100	Type 2 diabetes mellitus with hyperosmolarity without nonketotic hyperglycemic-hyperosmolar coma (NKHHC)
E1101	Type 2 diabetes mellitus with hyperosmolarity with coma
E111	Type 2 diabetes mellitus with ketoacidosis
E1110	Type 2 diabetes mellitus with ketoacidosis without coma
E1111	Type 2 diabetes mellitus with ketoacidosis with coma
E112	Type 2 diabetes mellitus with kidney complications
E1121	Type 2 diabetes mellitus with diabetic nephropathy
E1122	Type 2 diabetes mellitus with diabetic chronic kidney disease
E1129	Type 2 diabetes mellitus with other diabetic kidney complication
E113	Type 2 diabetes mellitus with ophthalmic complications
E1131	Type 2 diabetes mellitus with unspecified diabetic retinopathy
E11311	Type 2 diabetes mellitus with unspecified diabetic retinopathy with macular edema
E11319	Type 2 diabetes mellitus with unspecified diabetic retinopathy without macular edema
E1132	Type 2 diabetes mellitus with mild nonproliferative diabetic retinopathy
E11321	Type 2 diabetes mellitus with mild nonproliferative diabetic retinopathy with macular edema
E113211	Type 2 diabetes mellitus with mild nonproliferative diabetic retinopathy with macular edema, right eye

E113212	Type 2 diabetes mellitus with mild nonproliferative diabetic retinopathy with macular edema, left eye
E113213	Type 2 diabetes mellitus with mild nonproliferative diabetic retinopathy with macular edema, bilateral
E113219	Type 2 diabetes mellitus with mild nonproliferative diabetic retinopathy with macular edema, unspecified eye
E11329	Type 2 diabetes mellitus with mild nonproliferative diabetic retinopathy without macular edema
E113291	Type 2 diabetes mellitus with mild nonproliferative diabetic retinopathy without macular edema, right eye
E113292	Type 2 diabetes mellitus with mild nonproliferative diabetic retinopathy without macular edema, left eye
E113293	Type 2 diabetes mellitus with mild nonproliferative diabetic retinopathy without macular edema, bilateral
E113299	Type 2 diabetes mellitus with mild nonproliferative diabetic retinopathy without macular edema, unspecified eye
E1133	Type 2 diabetes mellitus with moderate nonproliferative diabetic retinopathy
E11331	Type 2 diabetes mellitus with moderate nonproliferative diabetic retinopathy with macular edema
E113311	Type 2 diabetes mellitus with moderate nonproliferative diabetic retinopathy with macular edema, right eye
E113312	Type 2 diabetes mellitus with moderate nonproliferative diabetic retinopathy with macular edema, left eye
E113313	Type 2 diabetes mellitus with moderate nonproliferative diabetic retinopathy with macular edema, bilateral
E113319	Type 2 diabetes mellitus with moderate nonproliferative diabetic retinopathy with macular edema, unspecified eye
E11339	Type 2 diabetes mellitus with moderate nonproliferative diabetic retinopathy without macular edema
E113391	Type 2 diabetes mellitus with moderate nonproliferative diabetic retinopathy without macular edema, right eye
E113392	Type 2 diabetes mellitus with moderate nonproliferative diabetic retinopathy without macular edema, left eye
E113393	Type 2 diabetes mellitus with moderate nonproliferative diabetic retinopathy without macular edema, bilateral
E113399	Type 2 diabetes mellitus with moderate nonproliferative diabetic retinopathy without macular edema, unspecified eye
E1134	Type 2 diabetes mellitus with severe nonproliferative diabetic retinopathy
E11341	Type 2 diabetes mellitus with severe nonproliferative diabetic retinopathy with macular edema
E113411	Type 2 diabetes mellitus with severe nonproliferative diabetic retinopathy with macular edema, right eye
E113412	Type 2 diabetes mellitus with severe nonproliferative diabetic retinopathy with macular edema, left eye
E113413	Type 2 diabetes mellitus with severe nonproliferative diabetic retinopathy with macular edema, bilateral
E113419	Type 2 diabetes mellitus with severe nonproliferative diabetic retinopathy with macular edema, unspecified eye
E11349	Type 2 diabetes mellitus with severe nonproliferative diabetic retinopathy without macular edema
E113491	Type 2 diabetes mellitus with severe nonproliferative diabetic retinopathy without macular edema, right eye
E113492	Type 2 diabetes mellitus with severe nonproliferative diabetic retinopathy without macular edema, left eye
E113493	Type 2 diabetes mellitus with severe nonproliferative diabetic retinopathy without macular edema, bilateral
E113499	Type 2 diabetes mellitus with severe nonproliferative diabetic retinopathy without macular edema, unspecified eye
E1135	Type 2 diabetes mellitus with proliferative diabetic retinopathy
E11351	Type 2 diabetes mellitus with proliferative diabetic retinopathy with macular edema
E113511	Type 2 diabetes mellitus with proliferative diabetic retinopathy with macular edema, right eye
E113512	Type 2 diabetes mellitus with proliferative diabetic retinopathy with macular edema, left eye
E113513	Type 2 diabetes mellitus with proliferative diabetic retinopathy with macular edema, bilateral
E113519	Type 2 diabetes mellitus with proliferative diabetic retinopathy with macular edema, unspecified eye
E11352	Type 2 diabetes mellitus with proliferative diabetic retinopathy with traction retinal detachment involving the macula
E113521	Type 2 diabetes mellitus with proliferative diabetic retinopathy with traction retinal detachment involving the macula, right eye
E113522	Type 2 diabetes mellitus with proliferative diabetic retinopathy with traction retinal detachment involving the macula, left eye
E113523	Type 2 diabetes mellitus with proliferative diabetic retinopathy with traction retinal detachment involving the macula, bilateral
E113529	Type 2 diabetes mellitus with proliferative diabetic retinopathy with traction retinal detachment involving the macula, unspecified eye
E11353	Type 2 diabetes mellitus with proliferative diabetic retinopathy with traction retinal detachment not involving the macula

© 2022 Sharifi M et al. *JAMA Network Open*.

E113531	Type 2 diabetes mellitus with proliferative diabetic retinopathy with traction retinal detachment not involving the macula, right eye
E113532	Type 2 diabetes mellitus with proliferative diabetic retinopathy with traction retinal detachment not involving the macula, left eye
E113533	Type 2 diabetes mellitus with proliferative diabetic retinopathy with traction retinal detachment not involving the macula, bilateral
E113539	Type 2 diabetes mellitus with proliferative diabetic retinopathy with traction retinal detachment not involving the macula, unspecified eye
E11354	Type 2 diabetes mellitus with proliferative diabetic retinopathy with combined traction retinal detachment and rhegmatogenous retinal detachment
E113541	Type 2 diabetes mellitus with proliferative diabetic retinopathy with combined traction retinal detachment and rhegmatogenous retinal detachment, right eye
E113542	Type 2 diabetes mellitus with proliferative diabetic retinopathy with combined traction retinal detachment and rhegmatogenous retinal detachment, left eye
E113543	Type 2 diabetes mellitus with proliferative diabetic retinopathy with combined traction retinal detachment and rhegmatogenous retinal detachment, bilateral
E113549	Type 2 diabetes mellitus with proliferative diabetic retinopathy with combined traction retinal detachment and rhegmatogenous retinal detachment, unspecified eye
E11355	Type 2 diabetes mellitus with stable proliferative diabetic retinopathy
E113551	Type 2 diabetes mellitus with stable proliferative diabetic retinopathy, right eye
E113552	Type 2 diabetes mellitus with stable proliferative diabetic retinopathy, left eye
E113553	Type 2 diabetes mellitus with stable proliferative diabetic retinopathy, bilateral
E113559	Type 2 diabetes mellitus with stable proliferative diabetic retinopathy, unspecified eye
E11359	Type 2 diabetes mellitus with proliferative diabetic retinopathy without macular edema
E113591	Type 2 diabetes mellitus with proliferative diabetic retinopathy without macular edema, right eye
E113592	Type 2 diabetes mellitus with proliferative diabetic retinopathy without macular edema, left eye
E113593	Type 2 diabetes mellitus with proliferative diabetic retinopathy without macular edema, bilateral
E113599	Type 2 diabetes mellitus with proliferative diabetic retinopathy without macular edema, unspecified eye
E1136	Type 2 diabetes mellitus with diabetic cataract
E1137	Type 2 diabetes mellitus with diabetic macular edema, resolved following treatment
E1137X1	Type 2 diabetes mellitus with diabetic macular edema, resolved following treatment, right eye
E1137X2	Type 2 diabetes mellitus with diabetic macular edema, resolved following treatment, left eye
E1137X3	Type 2 diabetes mellitus with diabetic macular edema, resolved following treatment, bilateral
E1137X9	Type 2 diabetes mellitus with diabetic macular edema, resolved following treatment, unspecified eye
E1139	Type 2 diabetes mellitus with other diabetic ophthalmic complication
E114	Type 2 diabetes mellitus with neurological complications
E1140	Type 2 diabetes mellitus with diabetic neuropathy, unspecified
E1141	Type 2 diabetes mellitus with diabetic mononeuropathy
E1142	Type 2 diabetes mellitus with diabetic polyneuropathy
E1143	Type 2 diabetes mellitus with diabetic autonomic (poly)neuropathy
E1144	Type 2 diabetes mellitus with diabetic amyotrophy
E1149	Type 2 diabetes mellitus with other diabetic neurological complication
E115	Type 2 diabetes mellitus with circulatory complications
E1151	Type 2 diabetes mellitus with diabetic peripheral angiopathy without gangrene
E1152	Type 2 diabetes mellitus with diabetic peripheral angiopathy with gangrene
E1159	Type 2 diabetes mellitus with other circulatory complications
E116	Type 2 diabetes mellitus with other specified complications
E1161	Type 2 diabetes mellitus with diabetic arthropathy
E11610	Type 2 diabetes mellitus with diabetic neuropathic arthropathy
E11618	Type 2 diabetes mellitus with other diabetic arthropathy
E1162	Type 2 diabetes mellitus with skin complications
E11620	Type 2 diabetes mellitus with diabetic dermatitis
E11621	Type 2 diabetes mellitus with foot ulcer
E11622	Type 2 diabetes mellitus with other skin ulcer
E11628	Type 2 diabetes mellitus with other skin complications
E1163	Type 2 diabetes mellitus with oral complications
E11630	Type 2 diabetes mellitus with periodontal disease

© 2022 Sharifi M et al. *JAMA Network Open*.

E11638	Type 2 diabetes mellitus with other oral complications
E1164	Type 2 diabetes mellitus with hypoglycemia
E11641	Type 2 diabetes mellitus with hypoglycemia with coma
E11649	Type 2 diabetes mellitus with hypoglycemia without coma
E1165	Type 2 diabetes mellitus with hyperglycemia
E1169	Type 2 diabetes mellitus with other specified complication
E118	Type 2 diabetes mellitus with unspecified complications
E119	Type 2 diabetes mellitus without complications
E13	Other specified diabetes mellitus
E130	Other specified diabetes mellitus with hyperosmolarity
E1300	Other specified diabetes mellitus with hyperosmolarity without nonketotic hyperglycemic-hyperosmolar coma (NKHHC)
E1301	Other specified diabetes mellitus with hyperosmolarity with coma
E131	Other specified diabetes mellitus with ketoacidosis
E1310	Other specified diabetes mellitus with ketoacidosis without coma
E1311	Other specified diabetes mellitus with ketoacidosis with coma
E132	Other specified diabetes mellitus with kidney complications
E1321	Other specified diabetes mellitus with diabetic nephropathy
E1322	Other specified diabetes mellitus with diabetic chronic kidney disease
E1329	Other specified diabetes mellitus with other diabetic kidney complication
E133	Other specified diabetes mellitus with ophthalmic complications
E1331	Other specified diabetes mellitus with unspecified diabetic retinopathy
E13311	Other specified diabetes mellitus with unspecified diabetic retinopathy with macular edema
E13319	Other specified diabetes mellitus with unspecified diabetic retinopathy without macular edema
E1332	Other specified diabetes mellitus with mild nonproliferative diabetic retinopathy
E13321	Other specified diabetes mellitus with mild nonproliferative diabetic retinopathy with macular edema
E133211	Other specified diabetes mellitus with mild nonproliferative diabetic retinopathy with macular edema, right eye
E133212	Other specified diabetes mellitus with mild nonproliferative diabetic retinopathy with macular edema, left eye
E133213	Other specified diabetes mellitus with mild nonproliferative diabetic retinopathy with macular edema, bilateral
E133219	Other specified diabetes mellitus with mild nonproliferative diabetic retinopathy with macular edema, unspecified eye
E13329	Other specified diabetes mellitus with mild nonproliferative diabetic retinopathy without macular edema
E133291	Other specified diabetes mellitus with mild nonproliferative diabetic retinopathy without macular edema, right eye
E133292	Other specified diabetes mellitus with mild nonproliferative diabetic retinopathy without macular edema, left eye
E133293	Other specified diabetes mellitus with mild nonproliferative diabetic retinopathy without macular edema, bilateral
E133299	Other specified diabetes mellitus with mild nonproliferative diabetic retinopathy without macular edema, unspecified eye
E1333	Other specified diabetes mellitus with moderate nonproliferative diabetic retinopathy
E13331	Other specified diabetes mellitus with moderate nonproliferative diabetic retinopathy with macular edema
E133311	Other specified diabetes mellitus with moderate nonproliferative diabetic retinopathy with macular edema, right eye
E133312	Other specified diabetes mellitus with moderate nonproliferative diabetic retinopathy with macular edema, left eye
E133313	Other specified diabetes mellitus with moderate nonproliferative diabetic retinopathy with macular edema, bilateral
E133319	Other specified diabetes mellitus with moderate nonproliferative diabetic retinopathy with macular edema, unspecified eye
E13339	Other specified diabetes mellitus with moderate nonproliferative diabetic retinopathy without macular edema
E133391	Other specified diabetes mellitus with moderate nonproliferative diabetic retinopathy without macular edema, right eye

© 2022 Sharifi M et al. *JAMA Network Open*.

E133392	Other specified diabetes mellitus with moderate nonproliferative diabetic retinopathy without macular edema, left eye
E133393	Other specified diabetes mellitus with moderate nonproliferative diabetic retinopathy without macular edema, bilateral
E133399	Other specified diabetes mellitus with moderate nonproliferative diabetic retinopathy without macular edema, unspecified eye
E1334	Other specified diabetes mellitus with severe nonproliferative diabetic retinopathy
E13341	Other specified diabetes mellitus with severe nonproliferative diabetic retinopathy with macular edema
E133411	Other specified diabetes mellitus with severe nonproliferative diabetic retinopathy with macular edema, right eye
E133412	Other specified diabetes mellitus with severe nonproliferative diabetic retinopathy with macular edema, left eye
E133413	Other specified diabetes mellitus with severe nonproliferative diabetic retinopathy with macular edema, bilateral
E133419	Other specified diabetes mellitus with severe nonproliferative diabetic retinopathy with macular edema, unspecified eye
E13349	Other specified diabetes mellitus with severe nonproliferative diabetic retinopathy without macular edema
E133491	Other specified diabetes mellitus with severe nonproliferative diabetic retinopathy without macular edema, right eye
E133492	Other specified diabetes mellitus with severe nonproliferative diabetic retinopathy without macular edema, left eye
E133493	Other specified diabetes mellitus with severe nonproliferative diabetic retinopathy without macular edema, bilateral
E133499	Other specified diabetes mellitus with severe nonproliferative diabetic retinopathy without macular edema, unspecified eye
E1335	Other specified diabetes mellitus with proliferative diabetic retinopathy
E13351	Other specified diabetes mellitus with proliferative diabetic retinopathy with macular edema
E133511	Other specified diabetes mellitus with proliferative diabetic retinopathy with macular edema, right eye
E133512	Other specified diabetes mellitus with proliferative diabetic retinopathy with macular edema, left eye
E133513	Other specified diabetes mellitus with proliferative diabetic retinopathy with macular edema, bilateral
E133519	Other specified diabetes mellitus with proliferative diabetic retinopathy with macular edema, unspecified eye
E13352	Other specified diabetes mellitus with proliferative diabetic retinopathy with traction retinal detachment involving the macula
E133521	Other specified diabetes mellitus with proliferative diabetic retinopathy with traction retinal detachment involving the macula, right eye
E133522	Other specified diabetes mellitus with proliferative diabetic retinopathy with traction retinal detachment involving the macula, left eye
E133523	Other specified diabetes mellitus with proliferative diabetic retinopathy with traction retinal detachment involving the macula, bilateral
E133529	Other specified diabetes mellitus with proliferative diabetic retinopathy with traction retinal detachment involving the macula, unspecified eye
E13353	Other specified diabetes mellitus with proliferative diabetic retinopathy with traction retinal detachment not involving the macula
E133531	Other specified diabetes mellitus with proliferative diabetic retinopathy with traction retinal detachment not involving the macula, right eye
E133532	Other specified diabetes mellitus with proliferative diabetic retinopathy with traction retinal detachment not involving the macula, left eye
E133533	Other specified diabetes mellitus with proliferative diabetic retinopathy with traction retinal detachment not involving the macula, bilateral
E133539	Other specified diabetes mellitus with proliferative diabetic retinopathy with traction retinal detachment not involving the macula, unspecified eye
E13354	Other specified diabetes mellitus with proliferative diabetic retinopathy with combined traction retinal detachment and rhegmatogenous retinal detachment
E133541	Other specified diabetes mellitus with proliferative diabetic retinopathy with combined traction retinal detachment and rhegmatogenous retinal detachment, right eye
E133542	Other specified diabetes mellitus with proliferative diabetic retinopathy with combined traction retinal detachment and rhegmatogenous retinal detachment, left eye

© 2022 Sharifi M et al. *JAMA Network Open*.

E133543	Other specified diabetes mellitus with proliferative diabetic retinopathy with combined traction retinal detachment and rhegmatogenous retinal detachment, bilateral
E133549	Other specified diabetes mellitus with proliferative diabetic retinopathy with combined traction retinal detachment and rhegmatogenous retinal detachment, unspecified eye
E13355	Other specified diabetes mellitus with stable proliferative diabetic retinopathy
E133551	Other specified diabetes mellitus with stable proliferative diabetic retinopathy, right eye
E133552	Other specified diabetes mellitus with stable proliferative diabetic retinopathy, left eye
E133553	Other specified diabetes mellitus with stable proliferative diabetic retinopathy, bilateral
E133559	Other specified diabetes mellitus with stable proliferative diabetic retinopathy, unspecified eye
E13359	Other specified diabetes mellitus with proliferative diabetic retinopathy without macular edema
E133591	Other specified diabetes mellitus with proliferative diabetic retinopathy without macular edema, right eye
E133592	Other specified diabetes mellitus with proliferative diabetic retinopathy without macular edema, left eye
E133593	Other specified diabetes mellitus with proliferative diabetic retinopathy without macular edema, bilateral
E133599	Other specified diabetes mellitus with proliferative diabetic retinopathy without macular edema, unspecified eye
E1336	Other specified diabetes mellitus with diabetic cataract
E1337	Other specified diabetes mellitus with diabetic macular edema, resolved following treatment
E1337X1	Other specified diabetes mellitus with diabetic macular edema, resolved following treatment, right eye
E1337X2	Other specified diabetes mellitus with diabetic macular edema, resolved following treatment, left eye
E1337X3	Other specified diabetes mellitus with diabetic macular edema, resolved following treatment, bilateral
E1337X9	Other specified diabetes mellitus with diabetic macular edema, resolved following treatment, unspecified eye
E1339	Other specified diabetes mellitus with other diabetic ophthalmic complication
E134	Other specified diabetes mellitus with neurological complications
E1340	Other specified diabetes mellitus with diabetic neuropathy, unspecified
E1341	Other specified diabetes mellitus with diabetic mononeuropathy
E1342	Other specified diabetes mellitus with diabetic polyneuropathy
E1343	Other specified diabetes mellitus with diabetic autonomic (poly)neuropathy
E1344	Other specified diabetes mellitus with diabetic amyotrophy
E1349	Other specified diabetes mellitus with other diabetic neurological complication
E135	Other specified diabetes mellitus with circulatory complications
E1351	Other specified diabetes mellitus with diabetic peripheral angiopathy without gangrene
E1352	Other specified diabetes mellitus with diabetic peripheral angiopathy with gangrene
E1359	Other specified diabetes mellitus with other circulatory complications
E136	Other specified diabetes mellitus with other specified complications
E1361	Other specified diabetes mellitus with diabetic arthropathy
E13610	Other specified diabetes mellitus with diabetic neuropathic arthropathy
E13618	Other specified diabetes mellitus with other diabetic arthropathy
E1362	Other specified diabetes mellitus with skin complications
E13620	Other specified diabetes mellitus with diabetic dermatitis
E13621	Other specified diabetes mellitus with foot ulcer
E13622	Other specified diabetes mellitus with other skin ulcer
E13628	Other specified diabetes mellitus with other skin complications
E1363	Other specified diabetes mellitus with oral complications
E13630	Other specified diabetes mellitus with periodontal disease
E13638	Other specified diabetes mellitus with other oral complications
E1364	Other specified diabetes mellitus with hypoglycemia
E13641	Other specified diabetes mellitus with hypoglycemia with coma
E13649	Other specified diabetes mellitus with hypoglycemia without coma
E1365	Other specified diabetes mellitus with hyperglycemia
E1369	Other specified diabetes mellitus with other specified complication
E138	Other specified diabetes mellitus with unspecified complications
E139	Other specified diabetes mellitus without complications
E15	Nondiabetic hypoglycemic coma
E16	Other disorders of pancreatic internal secretion
E160	Drug-induced hypoglycemia without coma
E161	Other hypoglycemia

© 2022 Sharifi M et al. *JAMA Network Open*.

E162	Hypoglycemia, unspecified
E163	Increased secretion of glucagon
E164	Increased secretion of gastrin
E168	Other specified disorders of pancreatic internal secretion
E169	Disorder of pancreatic internal secretion, unspecified
E20	Hypoparathyroidism
E200	Idiopathic hypoparathyroidism
E201	Pseudohypoparathyroidism
E208	Other hypoparathyroidism
E209	Hypoparathyroidism, unspecified
E21	Hyperparathyroidism and other disorders of parathyroid gland
E210	Primary hyperparathyroidism
E211	Secondary hyperparathyroidism, not elsewhere classified
E212	Other hyperparathyroidism
E213	Hyperparathyroidism, unspecified
E214	Other specified disorders of parathyroid gland
E215	Disorder of parathyroid gland, unspecified
E22	Hyperfunction of pituitary gland
E220	Acromegaly and pituitary gigantism
E221	Hyperprolactinemia
E222	Syndrome of inappropriate secretion of antidiuretic hormone
E228	Other hyperfunction of pituitary gland
E229	Hyperfunction of pituitary gland, unspecified
E23	Hypofunction and other disorders of the pituitary gland
E230	Hypopituitarism
E231	Drug-induced hypopituitarism
E232	Diabetes insipidus
E233	Hypothalamic dysfunction, not elsewhere classified
E236	Other disorders of pituitary gland
E237	Disorder of pituitary gland, unspecified
E24	Cushing's syndrome
E240	Pituitary-dependent Cushing's disease
E241	Nelson's syndrome
E242	Drug-induced Cushing's syndrome
E243	Ectopic ACTH syndrome
E244	Alcohol-induced pseudo-Cushing's syndrome
E248	Other Cushing's syndrome
E249	Cushing's syndrome, unspecified
E25	Adrenogenital disorders
E250	Congenital adrenogenital disorders associated with enzyme deficiency
E258	Other adrenogenital disorders
E259	Adrenogenital disorder, unspecified
E26	Hyperaldosteronism
E260	Primary hyperaldosteronism
E2601	Conn's syndrome
E2602	Glucocorticoid-remediable aldosteronism
E2609	Other primary hyperaldosteronism
E261	Secondary hyperaldosteronism
E268	Other hyperaldosteronism
E2681	Bartter's syndrome
E2689	Other hyperaldosteronism
E269	Hyperaldosteronism, unspecified
E27	Other disorders of adrenal gland
E270	Other adrenocortical overactivity
E271	Primary adrenocortical insufficiency
E272	Addisonian crisis
E273	Drug-induced adrenocortical insufficiency

© 2022 Sharifi M et al. *JAMA Network Open*.

E274	Other and unspecified adrenocortical insufficiency
E2740	Unspecified adrenocortical insufficiency
E2749	Other adrenocortical insufficiency
E275	Adrenomedullary hyperfunction
E278	Other specified disorders of adrenal gland
E279	Disorder of adrenal gland, unspecified
E28	Ovarian dysfunction
E280	Estrogen excess
E281	Androgen excess
E282	Polycystic ovarian syndrome
E283	Primary ovarian failure
E2831	Premature menopause
E28310	Symptomatic premature menopause
E28319	Asymptomatic premature menopause
E2839	Other primary ovarian failure
E288	Other ovarian dysfunction
E289	Ovarian dysfunction, unspecified
E29	Testicular dysfunction
E290	Testicular hyperfunction
E291	Testicular hypofunction
E298	Other testicular dysfunction
E299	Testicular dysfunction, unspecified
E30	Disorders of puberty, not elsewhere classified
E300	Delayed puberty
E301	Precocious puberty
E308	Other disorders of puberty
E309	Disorder of puberty, unspecified
E31	Polyglandular dysfunction
E310	Autoimmune polyglandular failure
E311	Polyglandular hyperfunction
E312	Multiple endocrine neoplasia [MEN] syndromes
E3120	Multiple endocrine neoplasia [MEN] syndrome, unspecified
E3121	Multiple endocrine neoplasia [MEN] type I
E3122	Multiple endocrine neoplasia [MEN] type IIA
E3123	Multiple endocrine neoplasia [MEN] type IIB
E318	Other polyglandular dysfunction
E319	Polyglandular dysfunction, unspecified
E32	Diseases of thymus
E320	Persistent hyperplasia of thymus
E321	Abscess of thymus
E328	Other diseases of thymus
E329	Disease of thymus, unspecified
E34	Other endocrine disorders
E340	Carcinoid syndrome
E341	Other hypersecretion of intestinal hormones
E342	Ectopic hormone secretion, not elsewhere classified
E343	Short stature due to endocrine disorder
E344	Constitutional tall stature
E345	Androgen insensitivity syndrome
E3450	Androgen insensitivity syndrome, unspecified
E3451	Complete androgen insensitivity syndrome
E3452	Partial androgen insensitivity syndrome
E348	Other specified endocrine disorders
E349	Endocrine disorder, unspecified
E35	Disorders of endocrine glands in diseases classified elsewhere
E36	Intraoperative complications of endocrine system

© 2022 Sharifi M et al. *JAMA Network Open*.

E360	Intraoperative hemorrhage and hematoma of an endocrine system organ or structure complicating a procedure
E3601	Intraoperative hemorrhage and hematoma of an endocrine system organ or structure complicating an endocrine system procedure
E3602	Intraoperative hemorrhage and hematoma of an endocrine system organ or structure complicating other procedure
E361	Accidental puncture and laceration of an endocrine system organ or structure during a procedure
E3611	Accidental puncture and laceration of an endocrine system organ or structure during an endocrine system procedure
E3612	Accidental puncture and laceration of an endocrine system organ or structure during other procedure
E368	Other intraoperative complications of endocrine system
E40	Kwashiorkor
E41	Nutritional marasmus
E42	Marasmic kwashiorkor
E43	Unspecified severe protein-calorie malnutrition
E44	Protein-calorie malnutrition of moderate and mild degree
E440	Moderate protein-calorie malnutrition
E441	Mild protein-calorie malnutrition
E45	Retarded development following protein-calorie malnutrition
E46	Unspecified protein-calorie malnutrition
F50	Eating disorders
F500	Anorexia nervosa
F5000	Anorexia nervosa, unspecified
F5001	Anorexia nervosa, restricting type
F5002	Anorexia nervosa, binge eating/purging type
F502	Bulimia nervosa
F508	Other eating disorders
F5081	Binge eating disorder
F5082	Avoidant/restrictive food intake disorder
F5089	Other specified eating disorder
F509	Eating disorder, unspecified
K50	Crohn's disease [regional enteritis]
K500	Crohn's disease of small intestine
K5000	Crohn's disease of small intestine without complications
K5001	Crohn's disease of small intestine with complications
K50011	Crohn's disease of small intestine with rectal bleeding
K50012	Crohn's disease of small intestine with intestinal obstruction
K50013	Crohn's disease of small intestine with fistula
K50014	Crohn's disease of small intestine with abscess
K50018	Crohn's disease of small intestine with other complication
K50019	Crohn's disease of small intestine with unspecified complications
K501	Crohn's disease of large intestine
K5010	Crohn's disease of large intestine without complications
K5011	Crohn's disease of large intestine with complications
K50111	Crohn's disease of large intestine with rectal bleeding
K50112	Crohn's disease of large intestine with intestinal obstruction
K50113	Crohn's disease of large intestine with fistula
K50114	Crohn's disease of large intestine with abscess
K50118	Crohn's disease of large intestine with other complication
K50119	Crohn's disease of large intestine with unspecified complications
K508	Crohn's disease of both small and large intestine
K5080	Crohn's disease of both small and large intestine without complications
K5081	Crohn's disease of both small and large intestine with complications
K50811	Crohn's disease of both small and large intestine with rectal bleeding
K50812	Crohn's disease of both small and large intestine with intestinal obstruction
K50813	Crohn's disease of both small and large intestine with fistula
K50814	Crohn's disease of both small and large intestine with abscess

© 2022 Sharifi M et al. *JAMA Network Open*.

K50818	Crohn's disease of both small and large intestine with other complication
K50819	Crohn's disease of both small and large intestine with unspecified complications
K509	Crohn's disease, unspecified
K5090	Crohn's disease, unspecified, without complications
K5091	Crohn's disease, unspecified, with complications
K50911	Crohn's disease, unspecified, with rectal bleeding
K50912	Crohn's disease, unspecified, with intestinal obstruction
K50913	Crohn's disease, unspecified, with fistula
K50914	Crohn's disease, unspecified, with abscess
K50918	Crohn's disease, unspecified, with other complication
K50919	Crohn's disease, unspecified, with unspecified complications
K51	Ulcerative colitis
K510	Ulcerative (chronic) pancolitis
K5100	Ulcerative (chronic) pancolitis without complications
K5101	Ulcerative (chronic) pancolitis with complications
K51011	Ulcerative (chronic) pancolitis with rectal bleeding
K51012	Ulcerative (chronic) pancolitis with intestinal obstruction
K51013	Ulcerative (chronic) pancolitis with fistula
K51014	Ulcerative (chronic) pancolitis with abscess
K51018	Ulcerative (chronic) pancolitis with other complication
K51019	Ulcerative (chronic) pancolitis with unspecified complications
K512	Ulcerative (chronic) proctitis
K5120	Ulcerative (chronic) proctitis without complications
K5121	Ulcerative (chronic) proctitis with complications
K51211	Ulcerative (chronic) proctitis with rectal bleeding
K51212	Ulcerative (chronic) proctitis with intestinal obstruction
K51213	Ulcerative (chronic) proctitis with fistula
K51214	Ulcerative (chronic) proctitis with abscess
K51218	Ulcerative (chronic) proctitis with other complication
K51219	Ulcerative (chronic) proctitis with unspecified complications
K513	Ulcerative (chronic) rectosigmoiditis
K5130	Ulcerative (chronic) rectosigmoiditis without complications
K5131	Ulcerative (chronic) rectosigmoiditis with complications
K51311	Ulcerative (chronic) rectosigmoiditis with rectal bleeding
K51312	Ulcerative (chronic) rectosigmoiditis with intestinal obstruction
K51313	Ulcerative (chronic) rectosigmoiditis with fistula
K51314	Ulcerative (chronic) rectosigmoiditis with abscess
K51318	Ulcerative (chronic) rectosigmoiditis with other complication
K51319	Ulcerative (chronic) rectosigmoiditis with unspecified complications
K514	Inflammatory polyps of colon
K5140	Inflammatory polyps of colon without complications
K5141	Inflammatory polyps of colon with complications
K51411	Inflammatory polyps of colon with rectal bleeding
K51412	Inflammatory polyps of colon with intestinal obstruction
K51413	Inflammatory polyps of colon with fistula
K51414	Inflammatory polyps of colon with abscess
K51418	Inflammatory polyps of colon with other complication
K51419	Inflammatory polyps of colon with unspecified complications
K515	Left sided colitis
K5150	Left sided colitis without complications
K5151	Left sided colitis with complications
K51511	Left sided colitis with rectal bleeding
K51512	Left sided colitis with intestinal obstruction
K51513	Left sided colitis with fistula
K51514	Left sided colitis with abscess
K51518	Left sided colitis with other complication
K51519	Left sided colitis with unspecified complications

© 2022 Sharifi M et al. *JAMA Network Open*.

K518	Other ulcerative colitis
K5180	Other ulcerative colitis without complications
K5181	Other ulcerative colitis with complications
K51811	Other ulcerative colitis with rectal bleeding
K51812	Other ulcerative colitis with intestinal obstruction
K51813	Other ulcerative colitis with fistula
K51814	Other ulcerative colitis with abscess
K51818	Other ulcerative colitis with other complication
K51819	Other ulcerative colitis with unspecified complications
K519	Ulcerative colitis, unspecified
K5190	Ulcerative colitis, unspecified, without complications
K5191	Ulcerative colitis, unspecified, with complications
K51911	Ulcerative colitis, unspecified with rectal bleeding
K51912	Ulcerative colitis, unspecified with intestinal obstruction
K51913	Ulcerative colitis, unspecified with fistula
K51914	Ulcerative colitis, unspecified with abscess
K51918	Ulcerative colitis, unspecified with other complication
K51919	Ulcerative colitis, unspecified with unspecified complications
K900	Celiac disease
L405	Arthropathic psoriasis
L4050	Arthropathic psoriasis, unspecified
L4051	Distal interphalangeal psoriatic arthropathy
L4052	Psoriatic arthritis mutilans
L4053	Psoriatic spondylitis
L4054	Psoriatic juvenile arthropathy
L4059	Other psoriatic arthropathy
M08	Juvenile arthritis
M080	Unspecified juvenile rheumatoid arthritis
M0800	Unspecified juvenile rheumatoid arthritis of unspecified site
M0801	Unspecified juvenile rheumatoid arthritis, shoulder
M08011	Unspecified juvenile rheumatoid arthritis, right shoulder
M08012	Unspecified juvenile rheumatoid arthritis, left shoulder
M08019	Unspecified juvenile rheumatoid arthritis, unspecified shoulder
M0802	Unspecified juvenile rheumatoid arthritis of elbow
M08021	Unspecified juvenile rheumatoid arthritis, right elbow
M08022	Unspecified juvenile rheumatoid arthritis, left elbow
M08029	Unspecified juvenile rheumatoid arthritis, unspecified elbow
M0803	Unspecified juvenile rheumatoid arthritis, wrist
M08031	Unspecified juvenile rheumatoid arthritis, right wrist
M08032	Unspecified juvenile rheumatoid arthritis, left wrist
M08039	Unspecified juvenile rheumatoid arthritis, unspecified wrist
M0804	Unspecified juvenile rheumatoid arthritis, hand
M08041	Unspecified juvenile rheumatoid arthritis, right hand
M08042	Unspecified juvenile rheumatoid arthritis, left hand
M08049	Unspecified juvenile rheumatoid arthritis, unspecified hand
M0805	Unspecified juvenile rheumatoid arthritis, hip
M08051	Unspecified juvenile rheumatoid arthritis, right hip
M08052	Unspecified juvenile rheumatoid arthritis, left hip
M08059	Unspecified juvenile rheumatoid arthritis, unspecified hip
M0806	Unspecified juvenile rheumatoid arthritis, knee
M08061	Unspecified juvenile rheumatoid arthritis, right knee
M08062	Unspecified juvenile rheumatoid arthritis, left knee
M08069	Unspecified juvenile rheumatoid arthritis, unspecified knee
M0807	Unspecified juvenile rheumatoid arthritis, ankle and foot
M08071	Unspecified juvenile rheumatoid arthritis, right ankle and foot
M08072	Unspecified juvenile rheumatoid arthritis, left ankle and foot
M08079	Unspecified juvenile rheumatoid arthritis, unspecified ankle and foot

© 2022 Sharifi M et al. *JAMA Network Open*.

M0808	Unspecified juvenile rheumatoid arthritis, vertebrae
M0809	Unspecified juvenile rheumatoid arthritis, multiple sites
M080A	Unspecified juvenile rheumatoid arthritis, other specified site
M081	Juvenile ankylosing spondylitis
M082	Juvenile rheumatoid arthritis with systemic onset
M0820	Juvenile rheumatoid arthritis with systemic onset, unspecified site
M0821	Juvenile rheumatoid arthritis with systemic onset, shoulder
M08211	Juvenile rheumatoid arthritis with systemic onset, right shoulder
M08212	Juvenile rheumatoid arthritis with systemic onset, left shoulder
M08219	Juvenile rheumatoid arthritis with systemic onset, unspecified shoulder
M0822	Juvenile rheumatoid arthritis with systemic onset, elbow
M08221	Juvenile rheumatoid arthritis with systemic onset, right elbow
M08222	Juvenile rheumatoid arthritis with systemic onset, left elbow
M08229	Juvenile rheumatoid arthritis with systemic onset, unspecified elbow
M0823	Juvenile rheumatoid arthritis with systemic onset, wrist
M08231	Juvenile rheumatoid arthritis with systemic onset, right wrist
M08232	Juvenile rheumatoid arthritis with systemic onset, left wrist
M08239	Juvenile rheumatoid arthritis with systemic onset, unspecified wrist
M0824	Juvenile rheumatoid arthritis with systemic onset, hand
M08241	Juvenile rheumatoid arthritis with systemic onset, right hand
M08242	Juvenile rheumatoid arthritis with systemic onset, left hand
M08249	Juvenile rheumatoid arthritis with systemic onset, unspecified hand
M0825	Juvenile rheumatoid arthritis with systemic onset, hip
M08251	Juvenile rheumatoid arthritis with systemic onset, right hip
M08252	Juvenile rheumatoid arthritis with systemic onset, left hip
M08259	Juvenile rheumatoid arthritis with systemic onset, unspecified hip
M0826	Juvenile rheumatoid arthritis with systemic onset, knee
M08261	Juvenile rheumatoid arthritis with systemic onset, right knee
M08262	Juvenile rheumatoid arthritis with systemic onset, left knee
M08269	Juvenile rheumatoid arthritis with systemic onset, unspecified knee
M0827	Juvenile rheumatoid arthritis with systemic onset, ankle and foot
M08271	Juvenile rheumatoid arthritis with systemic onset, right ankle and foot
M08272	Juvenile rheumatoid arthritis with systemic onset, left ankle and foot
M08279	Juvenile rheumatoid arthritis with systemic onset, unspecified ankle and foot
M0828	Juvenile rheumatoid arthritis with systemic onset, vertebrae
M0829	Juvenile rheumatoid arthritis with systemic onset, multiple sites
M082A	Juvenile rheumatoid arthritis with systemic onset, other specified site
M083	Juvenile rheumatoid polyarthritis (seronegative)
M084	Pauciarticular juvenile rheumatoid arthritis
M0840	Pauciarticular juvenile rheumatoid arthritis, unspecified site
M0841	Pauciarticular juvenile rheumatoid arthritis, shoulder
M08411	Pauciarticular juvenile rheumatoid arthritis, right shoulder
M08412	Pauciarticular juvenile rheumatoid arthritis, left shoulder
M08419	Pauciarticular juvenile rheumatoid arthritis, unspecified shoulder
M0842	Pauciarticular juvenile rheumatoid arthritis, elbow
M08421	Pauciarticular juvenile rheumatoid arthritis, right elbow
M08422	Pauciarticular juvenile rheumatoid arthritis, left elbow
M08429	Pauciarticular juvenile rheumatoid arthritis, unspecified elbow
M0843	Pauciarticular juvenile rheumatoid arthritis, wrist
M08431	Pauciarticular juvenile rheumatoid arthritis, right wrist
M08432	Pauciarticular juvenile rheumatoid arthritis, left wrist
M08439	Pauciarticular juvenile rheumatoid arthritis, unspecified wrist
M0844	Pauciarticular juvenile rheumatoid arthritis, hand
M08441	Pauciarticular juvenile rheumatoid arthritis, right hand
M08442	Pauciarticular juvenile rheumatoid arthritis, left hand
M08449	Pauciarticular juvenile rheumatoid arthritis, unspecified hand
M0845	Pauciarticular juvenile rheumatoid arthritis, hip

© 2022 Sharifi M et al. *JAMA Network Open*.

M08451	Pauciarticular juvenile rheumatoid arthritis, right hip
M08452	Pauciarticular juvenile rheumatoid arthritis, left hip
M08459	Pauciarticular juvenile rheumatoid arthritis, unspecified hip
M0846	Pauciarticular juvenile rheumatoid arthritis, knee
M08461	Pauciarticular juvenile rheumatoid arthritis, right knee
M08462	Pauciarticular juvenile rheumatoid arthritis, left knee
M08469	Pauciarticular juvenile rheumatoid arthritis, unspecified knee
M0847	Pauciarticular juvenile rheumatoid arthritis, ankle and foot
M08471	Pauciarticular juvenile rheumatoid arthritis, right ankle and foot
M08472	Pauciarticular juvenile rheumatoid arthritis, left ankle and foot
M08479	Pauciarticular juvenile rheumatoid arthritis, unspecified ankle and foot
M0848	Pauciarticular juvenile rheumatoid arthritis, vertebrae
M084A	Pauciarticular juvenile rheumatoid arthritis, other specified site
M088	Other juvenile arthritis
M0880	Other juvenile arthritis, unspecified site
M0881	Other juvenile arthritis, shoulder
M08811	Other juvenile arthritis, right shoulder
M08812	Other juvenile arthritis, left shoulder
M08819	Other juvenile arthritis, unspecified shoulder
M0882	Other juvenile arthritis, elbow
M08821	Other juvenile arthritis, right elbow
M08822	Other juvenile arthritis, left elbow
M08829	Other juvenile arthritis, unspecified elbow
M0883	Other juvenile arthritis, wrist
M08831	Other juvenile arthritis, right wrist
M08832	Other juvenile arthritis, left wrist
M08839	Other juvenile arthritis, unspecified wrist
M0884	Other juvenile arthritis, hand
M08841	Other juvenile arthritis, right hand
M08842	Other juvenile arthritis, left hand
M08849	Other juvenile arthritis, unspecified hand
M0885	Other juvenile arthritis, hip
M08851	Other juvenile arthritis, right hip
M08852	Other juvenile arthritis, left hip
M08859	Other juvenile arthritis, unspecified hip
M0886	Other juvenile arthritis, knee
M08861	Other juvenile arthritis, right knee
M08862	Other juvenile arthritis, left knee
M08869	Other juvenile arthritis, unspecified knee
M0887	Other juvenile arthritis, ankle and foot
M08871	Other juvenile arthritis, right ankle and foot
M08872	Other juvenile arthritis, left ankle and foot
M08879	Other juvenile arthritis, unspecified ankle and foot
M0888	Other juvenile arthritis, other specified site
M0889	Other juvenile arthritis, multiple sites
M089	Juvenile arthritis, unspecified
M0890	Juvenile arthritis, unspecified, unspecified site
M0891	Juvenile arthritis, unspecified, shoulder
M08911	Juvenile arthritis, unspecified, right shoulder
M08912	Juvenile arthritis, unspecified, left shoulder
M08919	Juvenile arthritis, unspecified, unspecified shoulder
M0892	Juvenile arthritis, unspecified, elbow
M08921	Juvenile arthritis, unspecified, right elbow
M08922	Juvenile arthritis, unspecified, left elbow
M08929	Juvenile arthritis, unspecified, unspecified elbow
M0893	Juvenile arthritis, unspecified, wrist
M08931	Juvenile arthritis, unspecified, right wrist

© 2022 Sharifi M et al. *JAMA Network Open*.

M08932	Juvenile arthritis, unspecified, left wrist
M08939	Juvenile arthritis, unspecified, unspecified wrist
M0894	Juvenile arthritis, unspecified, hand
M08941	Juvenile arthritis, unspecified, right hand
M08942	Juvenile arthritis, unspecified, left hand
M08949	Juvenile arthritis, unspecified, unspecified hand
M0895	Juvenile arthritis, unspecified, hip
M08951	Juvenile arthritis, unspecified, right hip
M08952	Juvenile arthritis, unspecified, left hip
M08959	Juvenile arthritis, unspecified, unspecified hip
M0896	Juvenile arthritis, unspecified, knee
M08961	Juvenile arthritis, unspecified, right knee
M08962	Juvenile arthritis, unspecified, left knee
M08969	Juvenile arthritis, unspecified, unspecified knee
M0897	Juvenile arthritis, unspecified, ankle and foot
M08971	Juvenile arthritis, unspecified, right ankle and foot
M08972	Juvenile arthritis, unspecified, left ankle and foot
M08979	Juvenile arthritis, unspecified, unspecified ankle and foot
M0898	Juvenile arthritis, unspecified, vertebrae
M0899	Juvenile arthritis, unspecified, multiple sites
M089A	Juvenile arthritis, unspecified, other specified site
M30	Polyarteritis nodosa and related conditions
M300	Polyarteritis nodosa
M301	Polyarteritis with lung involvement [Churg-Strauss]
M302	Juvenile polyarteritis
M303	Mucocutaneous lymph node syndrome [Kawasaki]
M308	Other conditions related to polyarteritis nodosa
M31	Other necrotizing vasculopathies
M310	Hypersensitivity angiitis
M311	Thrombotic microangiopathy
M312	Lethal midline granuloma
M313	Wegener's granulomatosis
M3130	Wegener's granulomatosis without renal involvement
M3131	Wegener's granulomatosis with renal involvement
M314	Aortic arch syndrome [Takayasu]
M315	Giant cell arteritis with polymyalgia rheumatica
M316	Other giant cell arteritis
M317	Microscopic polyangiitis
M318	Other specified necrotizing vasculopathies
M319	Necrotizing vasculopathy, unspecified
M32	Systemic lupus erythematosus (SLE)
M320	Drug-induced systemic lupus erythematosus
M321	Systemic lupus erythematosus with organ or system involvement
M3210	Systemic lupus erythematosus, organ or system involvement unspecified
M3211	Endocarditis in systemic lupus erythematosus
M3212	Pericarditis in systemic lupus erythematosus
M3213	Lung involvement in systemic lupus erythematosus
M3214	Glomerular disease in systemic lupus erythematosus
M3215	Tubulo-interstitial nephropathy in systemic lupus erythematosus
M3219	Other organ or system involvement in systemic lupus erythematosus
M328	Other forms of systemic lupus erythematosus
M329	Systemic lupus erythematosus, unspecified
M33	Dermatopolymyositis
M330	Juvenile dermatomyositis
M3300	Juvenile dermatomyositis, organ involvement unspecified
M3301	Juvenile dermatomyositis with respiratory involvement
M3302	Juvenile dermatomyositis with myopathy

© 2022 Sharifi M et al. *JAMA Network Open*.

M3303	Juvenile dermatomyositis without myopathy
M3309	Juvenile dermatomyositis with other organ involvement
M331	Other dermatomyositis
M3310	Other dermatomyositis, organ involvement unspecified
M3311	Other dermatomyositis with respiratory involvement
M3312	Other dermatomyositis with myopathy
M3313	Other dermatomyositis without myopathy
M3319	Other dermatomyositis with other organ involvement
M332	Polymyositis
M3320	Polymyositis, organ involvement unspecified
M3321	Polymyositis with respiratory involvement
M3322	Polymyositis with myopathy
M3329	Polymyositis with other organ involvement
M339	Dermatopolymyositis, unspecified
M3390	Dermatopolymyositis, unspecified, organ involvement unspecified
M3391	Dermatopolymyositis, unspecified with respiratory involvement
M3392	Dermatopolymyositis, unspecified with myopathy
M3393	Dermatopolymyositis, unspecified without myopathy
M3399	Dermatopolymyositis, unspecified with other organ involvement
M34	Systemic sclerosis [scleroderma]
M340	Progressive systemic sclerosis
M341	CR(E)ST syndrome
M342	Systemic sclerosis induced by drug and chemical
M348	Other forms of systemic sclerosis
M3481	Systemic sclerosis with lung involvement
M3482	Systemic sclerosis with myopathy
M3483	Systemic sclerosis with polyneuropathy
M3489	Other systemic sclerosis
M349	Systemic sclerosis, unspecified
M35	Other systemic involvement of connective tissue
M350	Sicca syndrome [Sjogren]
M3500	Sicca syndrome, unspecified
M3501	Sicca syndrome with keratoconjunctivitis
M3502	Sicca syndrome with lung involvement
M3503	Sicca syndrome with myopathy
M3504	Sicca syndrome with tubulo-interstitial nephropathy
M3509	Sicca syndrome with other organ involvement
M351	Other overlap syndromes
M352	Behcet's disease
M353	Polymyalgia rheumatica
M354	Diffuse (eosinophilic) fasciitis
M355	Multifocal fibrosclerosis
M356	Relapsing panniculitis [Weber-Christian]
M357	Hypermobility syndrome
M358	Other specified systemic involvement of connective tissue
M359	Systemic involvement of connective tissue, unspecified
M36	Systemic disorders of connective tissue in diseases classified elsewhere
M360	Dermato(poly)myositis in neoplastic disease
M361	Arthropathy in neoplastic disease
M362	Hemophilic arthropathy
M363	Arthropathy in other blood disorders
M364	Arthropathy in hypersensitivity reactions classified elsewhere
M368	Systemic disorders of connective tissue in other diseases classified elsewhere
M45	Ankylosing spondylitis
M450	Ankylosing spondylitis of multiple sites in spine
M451	Ankylosing spondylitis of occipito-atlanto-axial region
M452	Ankylosing spondylitis of cervical region

© 2022 Sharifi M et al. *JAMA Network Open*.

M453	Ankylosing spondylitis of cervicothoracic region
M454	Ankylosing spondylitis of thoracic region
M455	Ankylosing spondylitis of thoracolumbar region
M456	Ankylosing spondylitis lumbar region
M457	Ankylosing spondylitis of lumbosacral region
M458	Ankylosing spondylitis sacral and sacrococcygeal region
M459	Ankylosing spondylitis of unspecified sites in spine
N91	Absent, scanty and rare menstruation
N910	Primary amenorrhea
N911	Secondary amenorrhea
N912	Amenorrhea, unspecified
N913	Primary oligomenorrhea
N914	Secondary oligomenorrhea
N915	Oligomenorrhea, unspecified
N92	Excessive, frequent and irregular menstruation
N920	Excessive and frequent menstruation with regular cycle
N921	Excessive and frequent menstruation with irregular cycle
N922	Excessive menstruation at puberty
N923	Ovulation bleeding
N924	Excessive bleeding in the premenopausal period
N925	Other specified irregular menstruation
N926	Irregular menstruation, unspecified
Q900	Trisomy 21, nonmosaicism (meiotic nondisjunction)
Q901	Trisomy 21, mosaicism (mitotic nondisjunction)
Q902	Trisomy 21, translocation
Q909	Down syndrome, unspecified
Q91	Trisomy 18 and Trisomy 13
Q910	Trisomy 18, nonmosaicism (meiotic nondisjunction)
Q911	Trisomy 18, mosaicism (mitotic nondisjunction)
Q912	Trisomy 18, translocation
Q913	Trisomy 18, unspecified
Q914	Trisomy 13, nonmosaicism (meiotic nondisjunction)
Q915	Trisomy 13, mosaicism (mitotic nondisjunction)
Q916	Trisomy 13, translocation
Q917	Trisomy 13, unspecified
Q92	Other trisomies and partial trisomies of the autosomes, not elsewhere classified
Q920	Whole chromosome trisomy, nonmosaicism (meiotic nondisjunction)
Q921	Whole chromosome trisomy, mosaicism (mitotic nondisjunction)
Q922	Partial trisomy
Q925	Duplications with other complex rearrangements
Q926	Marker chromosomes
Q9261	Marker chromosomes in normal individual
Q9262	Marker chromosomes in abnormal individual
Q927	Triploidy and polyploidy
Q928	Other specified trisomies and partial trisomies of autosomes
Q929	Trisomy and partial trisomy of autosomes, unspecified
Q93	Monosomies and deletions from the autosomes, not elsewhere classified
Q930	Whole chromosome monosomy, nonmosaicism (meiotic nondisjunction)
Q931	Whole chromosome monosomy, mosaicism (mitotic nondisjunction)
Q932	Chromosome replaced with ring, dicentric or isochromosome
Q933	Deletion of short arm of chromosome 4
Q934	Deletion of short arm of chromosome 5
Q935	Other deletions of part of a chromosome
Q9351	Angelman syndrome
Q9359	Other deletions of part of a chromosome
Q937	Deletions with other complex rearrangements
Q938	Other deletions from the autosomes

© 2022 Sharifi M et al. *JAMA Network Open*.

Q9381	Velo-cardio-facial syndrome
Q9382	Williams syndrome
Q9388	Other microdeletions
Q9389	Other deletions from the autosomes
Q939	Deletion from autosomes, unspecified
Q95	Balanced rearrangements and structural markers, not elsewhere classified
Q950	Balanced translocation and insertion in normal individual
Q951	Chromosome inversion in normal individual
Q952	Balanced autosomal rearrangement in abnormal individual
Q953	Balanced sex/autosomal rearrangement in abnormal individual
Q955	Individual with autosomal fragile site
Q958	Other balanced rearrangements and structural markers
Q959	Balanced rearrangement and structural marker, unspecified
Q96	Turner's syndrome
Q960	Karyotype 45, X
Q961	Karyotype 46, X iso (Xq)
Q962	Karyotype 46, X with abnormal sex chromosome, except iso (Xq)
Q963	Mosaicism, 45, X/46, XX or XY
Q964	Mosaicism, 45, X/other cell line(s) with abnormal sex chromosome
Q968	Other variants of Turner's syndrome
Q969	Turner's syndrome, unspecified
Q97	Other sex chromosome abnormalities, female phenotype, not elsewhere classified
Q970	Karyotype 47, XXX
Q971	Female with more than three X chromosomes
Q972	Mosaicism, lines with various numbers of X chromosomes
Q973	Female with 46, XY karyotype
Q978	Other specified sex chromosome abnormalities, female phenotype
Q979	Sex chromosome abnormality, female phenotype, unspecified
Q98	Other sex chromosome abnormalities, male phenotype, not elsewhere classified
Q980	Klinefelter syndrome karyotype 47, XXY
Q981	Klinefelter syndrome, male with more than two X chromosomes
Q983	Other male with 46, XX karyotype
Q984	Klinefelter syndrome, unspecified
Q985	Karyotype 47, XYY
Q986	Male with structurally abnormal sex chromosome
Q987	Male with sex chromosome mosaicism
Q988	Other specified sex chromosome abnormalities, male phenotype
Q989	Sex chromosome abnormality, male phenotype, unspecified
Q99	Other chromosome abnormalities, not elsewhere classified
Q990	Chimera 46, XX/46, XY
Q991	46, XX true hermaphrodite
Q992	Fragile X chromosome
Q998	Other specified chromosome abnormalities
Q999	Chromosomal abnormality, unspecified

© 2022 Sharifi M et al. *JAMA Network Open*.
